# Ethnic Differences in Cancer Rates Among Adults With Type 2 Diabetes in New Zealand From 1994 to 2018

**DOI:** 10.1001/jamanetworkopen.2021.47171

**Published:** 2022-02-07

**Authors:** Dahai Yu, Zheng Wang, Yamei Cai, Kate McBride, Uchechukwu Levi Osuagwu, Karen Pickering, John Baker, Richard Cutfield, Brandon J. Orr-Walker, Gerhard Sundborn, Michael B. Jameson, Zhanzheng Zhao, David Simmons

**Affiliations:** 1 Department of Nephrology, The First Affiliated Hospital, Zhengzhou University, Zhengzhou, China; 2 Primary Care Centre Versus Arthritis, School of Medicine, Keele University, Keele, United Kingdom; 3 School of Medicine, Western Sydney University, Campbelltown, Sydney, New South Wales, Australia; 4 Diabetes Foundation Aotearoa, Otara, New Zealand; 5 Department of Diabetes and Endocrinology, Counties Manukau Health, South Auckland, New Zealand; 6 Department of Diabetes and Endocrinology, Waitemata District Health Board, Auckland, New Zealand; 7 Section of Pacific Health, The University of Auckland, Auckland, New Zealand; 8 Oncology Department, Waikato Hospital, Hamilton, New Zealand; 9 Waikato Clinical Campus, The University of Auckland, Hamilton, New Zealand

## Abstract

**Importance:**

People with type 2 diabetes have greater risk for some site-specific cancers, and risks of cancers differ among racial and ethnic groups in the general population of Aotearoa New Zealand. The extent of ethnic disparities in cancer risks among people with type 2 diabetes in New Zealand is unclear.

**Objective:**

To compare the risks of 21 common adult cancers among Māori, Pasifika, and New Zealand European individuals with type 2 diabetes in New Zealand from 1994 to 2018.

**Design, Setting, and Participants:**

This population-based, matched cohort study used data from the primary care audit program in Auckland, New Zealand, linked with national cancer, death, and hospitalization registration databases, collected from January 1, 1994, to July 31, 2018, with follow-up data obtained through December 31, 2019. Using a tapered matching method to balance potential confounders (sociodemographic characteristics, lifestyle, anthropometric and clinical measurements, treatments [antidiabetes, antihypertensive, lipid-lowering, and anticoagulant], period effects, and recorded duration of diabetes), comparative cohorts were formed between New Zealand European and Māori and New Zealand European and Pasifika individuals aged 18 years or older with type 2 diabetes. Sex-specific matched cohorts were formed for sex-specific cancers.

**Exposures:**

Māori, Pasifika, and New Zealand European (reference group) ethnicity.

**Main Outcomes and Measures:**

The incidence rates of 21 common cancers recorded in nationally linked databases between 1994 and 2018 were the main outcomes. Weighted Cox proportional hazards regression was used to assess ethnic differences in risk of each cancer.

**Results:**

A total of 33 524 adults were included: 15 469 New Zealand European (mean [SD] age, 61.6 [13.2] years; 8522 [55.1%] male), 6656 Māori (mean [SD] age, 51.2 [12.4] years; 3345 [50.3%] female), and 11 399 Pasifika (mean [SD] age, 52.8 [12.7] years; 5994 [52.6%] female) individuals. In the matched New Zealand European and Māori cohort (New Zealand European: 8361 individuals; mean [SD] age, 58.9 [12.9] years; 4595 [55.0%] male; Māori: 5039 individuals; mean [SD] age, 51.4 [12.3] years; 2542 [50.5%] male), significant differences between New Zealand European and Māori individuals were identified in the risk for 7 cancers. Compared with New Zealand European individuals, the hazard ratios (HRs) among Māori individuals were 15.36 (95% CI, 4.50-52.34) for thyroid cancer, 7.94 (95% CI, 1.57-40.24) for gallbladder cancer, 4.81 (95% CI, 1.08-21.42) for cervical cancer (females only), 1.97 (95% CI, 1.30-2.99) for lung cancer, 1.81 (95% CI, 1.08-3.03) for liver cancer, 0.56 (95% CI, 0.35-0.90) for colon cancer, and 0.11 (95% CI, 0.04-0.27) for malignant melanoma. In the matched New Zealand European and Pasifika cohort (New Zealand European: 9340 individuals; mean [SD] age, 60.6 [13.1] years; 4885 [52.3%] male; Pasifika: 8828 individuals; mean [SD] age, 53.1 [12.6] years; 4612 [52.2%] female), significant differences between New Zealand European and Pasifika individuals were identified for 6 cancers. Compared with New Zealand European individuals, HRs among Pasifika individuals were 25.10 (95% CI, 3.14-200.63) for gallbladder cancer, 4.47 (95% CI, 1.25-16.03) for thyroid cancer, 0.48 (95% CI, 0.30-0.78) for colon cancer, 0.21 (95% CI, 0.09-0.48) for rectal cancer, 0.21 (95% CI, 0.07-0.65) for malignant melanoma, and 0.01 (95% CI, 0.01-0.10) for bladder cancer.

**Conclusions and Relevance:**

In this cohort study, differences in the risk of 21 common cancers were found between New Zealand European, Māori, and Pasifika groups of adults with type 2 diabetes in New Zealand from 1994 to 2018. Research into the mechanisms underlying these differences as well as additional screening strategies (eg, for thyroid and gallbladder cancers) appear to be warranted.

## Key Points

**Question:**

Did risks for site-specific cancers differ among Māori, Pasifika, and New Zealand European adults with type 2 diabetes in New Zealand from 1994 to 2018?

**Findings:**

In this cohort study of 33 524 adults with type 2 diabetes in New Zealand from 1994 to 2018, compared with the New Zealand European cohort, the Māori cohort had significantly greater risk of liver, gallbladder, lung, thyroid, and cervical cancer and lower risk of colon cancer and malignant melanoma, and the Pasifika cohort had significantly greater risk of gallbladder and thyroid cancer and lower risk of colon, rectal, and bladder cancer and malignant melanoma.

**Meaning:**

These findings suggest that cancer prevention and screening activities that are specific to ethnic groups are needed among people with type 2 diabetes in New Zealand.

## Teaser

This cohort study of adults with type 2 diabetes from New Zealand compared the risk of 21 common cancers among individuals of Māori, Pasifika, and New Zealand European ethnicity between 1994 and 2018.

## Introduction

Diabetes and cancer are common noncommunicable diseases, and rates of these diseases have been increasing rapidly worldwide.^1^ In Aotearoa New Zealand in 2017, it was estimated that approximately 240 000 adults had diabetes and 24 453 had newly diagnosed cancer.^2^ Diabetes and cancer have been linked epidemiologically and biologically.^3^ Evidence has indicated that type 2 diabetes is associated with increased risk for several cancers (colorectal, breast, liver, pancreas, and bladder).^4,5^

In New Zealand, ethnic difference in risks for diabetes and cancer are well described.^6,7,8^ Māori and New Zealand Pasifika individuals have been found to have 2 to 3 times the risk of type 2 diabetes compared with New Zealand European individuals.^9^ In the general New Zealand population, it was estimated that between 2007 and 2016, 10 cancers were more common among Māori than non-Māori individuals.^2,10^ This is similar for Pasifika individuals, among whom cancer registration and mortality rates for more than 10 cancers are higher compared with those for non-Pasifika and non-Māori individuals.^2^ However, few studies^6^ have comprehensively investigated ethnic differences in the risk of cancer among people with type 2 diabetes after matching for population-level confounders such as demographic, socioeconomic, clinical, and treatment factors. We therefore compared the risk of 21 common cancers among people with type 2 diabetes in different ethnic groups (Māori vs New Zealand European and Pasifika vs New Zealand European) in New Zealand between 1994 and 2018 using a novel tapered matching method to establish comparative cohorts.

## Methods

### Data Sources

This cohort study used data from the Diabetes Care Support Service (DCSS). Established in 1991, with a run-in period until December 31, 1993, the DCSS audited general practice diabetes management in South, East, and West Auckland, New Zealand, until July 31, 2018.^11^ We linked the DCSS database with data from national cancer, death, hospitalization, pharmaceutical claim, and socioeconomic status databases to identify a cohort of patients aged 18 years or older with type 2 diabetes in Auckland. The resulting database included longitudinal, linked, deidentified data on demographics, diabetes clinical characteristics (including smoking status, diabetes duration, body mass index, blood pressure, hemoglobin A_1c_ levels, and lipid levels), and diabetes medications (antihypertensive, antidiabetes, statin, antiplatelet, and/or anticoagulant treatment). These data were validated through enumeration assessment and internal quality control policies with regular cross-checks by auditors, random and routine sampling and checking of data entry, and active data management (eg, queries, checking unusual numbers, ranking of columns, and duplicate checking).^11,12,13^ Pharmaceutical claims data included all prescriptions issued for patients and was used to cross-validate the DCSS prescription data. Only pharmaceutical claims data after 2006 were available for data linkage; National Health Index numbers were not universal before that time. Data for all patients from their first DCSS enrollment date (last enrollment, July 31, 2018) were included. The North Health Ethics Committee approved the DCSS for research purposes in 1992 and then as an ongoing audit in 1996. Ethics review was waived by the New Zealand Health and Disability Ethics Committees because anonymized data were used for this analysis. Written informed consent to participate was provided by an authorized signatory for each general practice. This study followed the Strengthening the Reporting of Observational Studies in Epidemiology (STROBE) reporting guideline.

### Population and Procedures

The study population included patients aged 18 years or older who had type 2 diabetes. Each participant’s enrollment date was their date of entry into the DCSS database. Patients were categorized into exposure groups by self-identified ethnicity (Māori, New Zealand European, or Pasifika). New Zealand European individuals were defined as those identifying as having any European ancestry. Māori (Indigenous Polynesian) individuals were defined as those identifying as having any Māori ancestry. Pasifika (93% Polynesian) individuals were defined as those identifying as having any Pasifika ancestry except Māori. The focal cohorts in this study included Māori and Pasifika patients; the control cohort included New Zealand European individuals because the study goal was to assess disparities in outcomes between the focal and control cohorts.

The area deprivation indicator, the NZDep2013 Index of Deprivation,^14^ was used to define socioeconomic status. The NZDep2013 provides an Index of Multiple Deprivation (IMD) score for each New Zealand meshblock (geographical unit containing a median of 81 people).^14^ Scores on the NZDep2013 scale of deprivation range from 1 to 10, with lower scores indicating less deprivation; the scale divides New Zealand into tenths of the distribution of the first principal component scores^14^ and was consistent with prior deprivation measures.^8^ To maintain statistical power, the IMD was redefined by recategorizing the NZDep2013 into 5 groups: IMD-1 (least deprived: NZDep2013 scores of 1-2); IMD-2, IMD-3, and IMD-4 (NZDep2013 scores of 3-4, 5-6, and 7-8, respectively); and IMD-5 (most deprived: NZDep2013 scores of 9-10). Type 2 diabetes within the DCSS database was defined by primary care medical record coding, with validation undertaken by trained diabetes auditors.

### Outcomes

Outcomes were defined as incident primary site-specific cancers. Incident cancer was defined as the first coded case of cancer at the site of interest in any of the linked databases at least 1 year after the enrollment date in the DCSS to rule out any potential information bias. Participants were excluded if their incident cancer diagnosis occurred within 1 year of enrollment. Participants were followed up from enrollment until an outcome of interest occurred, or for those without any outcome of interest, until December 31, 2019. All clinical events were defined by the primary *International Classification of Diseases, Ninth Revision (ICD-9)* and *International Statistical Classification of Diseases and Related Health Problems, Tenth Revision (ICD-10)* codes.

### Statistical Analysis

A tapered matching method was used to assess ethnic disparities in cancer risks between the focal and control groups because entropy balancing is the most popular approach.^15,16^ This involved incrementally matching the control cohort (New Zealand European) to the focal cohorts (Māori and Pasifika) using additional covariates and directly observing how the matched cohort changed in terms of hazard ratios (HRs) and in terms of unmatched covariates.

Before tapered matching and balancing, to reduce model dependence and the potential for irresolvable imbalances between the comparative cohorts, we used coarsened exact matching (CEM) to restrict the comparison of patients in comparative cohorts to areas of common support.^17^ This ensured sufficient overlap between comparative cohorts on key covariables and confounders (matching variables) for the 11 matching steps (eFigures 1-6 in the Supplement). The CEM algorithm is a monotonic imbalance-reducing matching method, which means the balance between the comparison groups is chosen by ex ante user choice rather than discovered through the usual process of “checking after the fact, tweaking the method, and repeatedly re-estimating.”^18^^(p1)^ Participants in comparison groups matched on step 11 were retained (eFigures 1-6 in the Supplement).

During the CEM, we restricted comparison between ethnic groups to areas of common support (ie, with sufficient overlap between 2 groups). After excluding participants with no areas of common support (eFigure 1 in the Supplement), we used entropy balancing, the most common tapered matching method,^15^ to minimize differences in the distribution of matching variables between comparison groups. Entropy balancing involves maximum entropy reweighting of the control group (in the present study, the New Zealand European cohort) by directly incorporating covariable balance into the weight function, in which the matched sample is reweighted in each matching step to key target moments (mean, variance, and skewness). For continuous matching variables, all 3 moments should be met; for binary variables, the only moment to meet is the mean because this is sufficient to ensure the match of higher moments (variance and skewness). All preprocessing (both CEM and entropy balancing) was performed without reference to outcomes.^19^

To compare sex-specific cancers (breast, cervical, and ovarian for women and prostate for men), 4 other comparison cohorts (New Zealand European women vs Māori women, New Zealand European women vs Pasifika women, New Zealand European men vs Māori men, and New Zealand European men vs Pasifika men) were matched using the same matching method (excluding sex as a matching variable) (eFigures 3-6 in the Supplement). Weighted Cox proportional hazards regression incorporating matching weights estimated from each matching step by entropy matching was applied in each matching step to provide an estimate of the relative risk of outcomes between comparison groups, with the New Zealand European group as the reference. Eleven matching steps were implemented that led to 11 models. Model 1 was weighted for age and sex; model 2, for all variables in model 1 plus IMD group; model 3, for all variables in model 2 plus smoking status; model 4, for all variables in model 3 plus body measurements (body mass index and systolic and diastolic blood pressure); model 5, for all variables in model 4 plus baseline hemoglobin A1c levels; model 6, for all variables in model 5 plus baseline lipid profiles (total cholesterol, low-density lipoprotein cholesterol, high-density lipoprotein cholesterol, and triglyceride levels); model 7, for all variables in model 6 plus baseline creatinine level; model 8, for all variables in model 7 plus antidiabetes treatments; model 9, for all variables in model 8 plus antihypertensive, lipid-lowering, and anticoagulant treatments; model 10, for all variables in model 9 plus entry cohorts; and model 11, for all variables in model 10 plus the duration of diabetes at study entry time. Analyses were conducted using Stata/MP, version 16.0 (StataCorp LLC). Statistical significance was set at 2-tailed *P* < .05.

## Results

We identified a total of 33 524 New Zealand adults diagnosed with type 2 diabetes between 1994 and 2018: 15 469 New Zealand European (mean [SD] age, 61.6 [13.2] years; 8522 [55.1%] male), 6656 Māori (mean [SD] age, 51.2 [12.4] years; 3345 [50.3%] female), and 11 399 Pasifika (mean [SD] age, 52.8 [12.7] years; 5994 [52.6%] female) individuals. The matched variables were different between the New Zealand European and Māori groups and between the New Zealand European and Pasifika groups (Table 1 and Table 2 and eFigure 7 in the Supplement). After CEM, 8361 New Zealand European individuals (mean [SD] age, 58.9 [12.9] years; 4595 [55.0%] male) were matched with 5039 Māori individuals (mean [SD] age, 51.4 [12.3] years; 2542 [50.5%] male) and 9340 New Zealand European individuals (mean [SD] age, 60.6 [13.1] years; 4885 [52.3%] male) were matched with 8828 Pasifika individuals (mean [SD] age, 53.1 [12.6] years; 4612 [52.2%] female) using the 11 matching steps (eFigures 1 and 2 in the Supplement). After CEM, matched variables tended to be more similar (Table 1 and Table 2 and eFigure 7 in the Supplement). Specifically, matched variables were most similar after the samples were weighted by entropy matching in terms of mean, variance, and skewness (Table 1 and Table 2 and eFigure 7 in the Supplement). The distributions of matching variables for sex-specific matched cohorts are presented in eTables 1 and 2 and eFigures 8 and 9 in the Supplement.

**Table 1.** Characteristics in the Comparison Cohorts of New Zealand European and Māori Individuals With Type 2 Diabetes in New Zealand From 1994 to 2018^a^

Characteristic	Unmatched cohorts			Cohorts after coarsened exact matching			Cohorts after entropy matching		
	New Zealand European (n = 15 469)	Māori (n = 6656)	*P* value	New Zealand European (n = 8361)	Māori (n = 5039)	*P* value	New Zealand European (n = 8361)	Māori (n = 5039)	*P* value
Age, mean (SD), y	61.6 (13.2)	51.2 (12.4)	<.001	58.9 (12.9)	51.4 (12.3)	<.001	50.7 (11.7)	50.7 (11.7)	>.99
Sex									
Female	6947 (44.9)	3345 (50.3)	<.001	3766 (45.0)	2497 (49.5)	<.001	51.5 (0.0)	51.5 (0.0)	>.99
Male	8522 (55.1)	3311 (49.7)	<.001	4595 (55.0)	2542 (50.5)	<.001	48.5 (0.0)	48.5 (0.0)	>.99
Enrollment cohort									
1994-1998	4716 (30.5)	1702 (25.6)	<.001	2648 (31.7)	1258 (25.0)	<.001	14.7 (0.0)	14.6 (0.0)	.86
1999-2003	4237 (27.4)	1594 (24.0)		2215 (26.5)	1236 (24.6)		22.2 (0.0)	22.6 (0.0)	
2004-2008	3054 (19.7)	1229 (18.5)		1583 (18.9)	916 (18.2)		24.0 (0.0)	23.4 (0.0)	
2009-2013	2426 (15.7)	1555 (23.4)		1426 (17.1)	1225 (24.4)		30.4 (0.0)	30.8 (0.0)	
2014-2018	1036 (6.7)	576 (8.7)		489 (5.9)	395 (7.9)		8.8 (0.0)	8.7 (0.0)	
Duration of diabetes, mean (SD), y	3.9 (1.1)	4.3 (1.4)	<.001	4.1 (1.1)	4.2 (1.3)	.33	3.7 (0.7)	3.7 (0.7)	>.99
IMD group (NZDep2013 scale score)									
1 (1 or 2)	2965 (19.2)	254 (3.8)	<.001	1425 (17.0)	199 (4.0)	<.001	3.8 (0.0)	3.9 (0.0)	.43
2 (3 or 4)	2651 (17.1)	493 (7.4)		1259 (15.1)	391 (7.8)		8.2 (0.0)	7.8 (0.0)	
3 (5 or 6)	2419 (15.6)	680 (10.2)		1067 (12.8)	472 (9.4)		8.7 (0.0)	9.4 (0.0)	
4 (7 or 8)	4056 (26.2)	1570 (23.6)		2528 (30.2)	1239 (24.6)		25.0 (0.0)	24.6 (0.0)	
5 (9 or 10)	3377 (21.8)	3659 (55.0)		2082 (24.9)	2739 (54.4)		54.2 (0.0)	54.3 (0.0)	
Smoking status									
Never	10 543 (68.2)	3096 (46.5)	<.001	5574 (66.7)	2458 (48.9)	<.001	41.6 (0.0)	41.6 (0.0)	>.99
Former	3226 (20.9)	1557 (23.4)		1799 (21.5)	1170 (23.3)		27.8 (0.0)	27.8 (0.0)	
Current	1700 (11.0)	2003 (30.1)		988 (11.8)	1402 (27.9)		30.6 (0.0)	30.6 (0.0)	
BMI, mean (SD)	31.2 (6.4)	35.8 (7.5)	<.001	32.5 (6.5)	36.3 (7.4)	<.001	36.3 (7.4)	36.3 (7.4)	>.99
Blood pressure, mean (SD), mm Hg									
Systolic	138 (18)	135 (19)	<.001	137 (17)	135 (18)	<.001	134 (18)	134 (18)	>.99
Diastolic	80 (10)	84 (12)	<.001	80 (10)	84 (11)	<.001	84 (11)	84 (11)	>.99
HbA_1c_ level, mean (SD), mmol/mol	54.2 (17.3)	64.9 (21.4)	<.001	55.4 (16.7)	63.9 (20.2)	<.001	63.9 (20.0)	63.9 (20.0)	>.99
Total cholesterol level, mean (SD), mmol/L	5.1 (1.2)	5.2 (1.2)	<.001	5.1 (1.1)	5.1 (1.1)	.98	5.1 (1.1)	5.1 (1.1)	>.99
Triglyceride level, mean (SD), mmol/L	2.2 (1.5)	2.6 (1.8)	<.001	2.2 (1.2)	2.5 (1.6)	<.001	2.5 (1.6)	2.5 (1.6)	>.99
LDL cholesterol level, mean (SD), mmol/L	2.5 (0.9)	2.6 (0.9)	<.001	2.5 (0.9)	2.6 (0.9)	<.001	2.6 (0.9)	2.6 (0.9)	>.99
HDL cholesterol level, mean (SD), mmol/L	1.2 (0.4)	1.1 (0.3)	<.001	1.2 (0.4)	1.1 (0.3)	<.001	1.1 (0.3)	1.1 (0.3)	>.99
Creatinine level, mean (SD), μmol/L	85.5 (18.4)	80.2 (18.9)	<.001	83.1 (17.9)	79.3 (18.1)	<.001	78.3 (18.1)	78.3 (18.1)	>.99
Antidiabetes treatment									
Oral drug and insulin	2398 (15.5)	1348 (20.3)	<.001	1742 (20.8)	1169 (23.2)	<.001	25.9 (0.0)	26.7 (0.0)	.39
Oral drug only	8640 (55.9)	3915 (58.8)		5106 (61.1)	3102 (61.7)		64.7 (0.0)	63.8 (0.0)	
Insulin only	571 (3.7)	205 (3.1)		309 (3.7)	137 (2.7)		2.1 (0.0)	2.1 (0.0)	
Antihypertensive treatment	10 676 (69.0)	4727 (71.0)	.003	6472 (77.4)	3927 (78.1)	.23	86.7 (0.0)	86.7 (0.0)	>.99
Statin treatment	8556 (55.3)	3890 (58.4)	<.001	5424 (64.9)	3320 (66.0)	.18	81.9 (0.0)	81.9 (0.0)	>.99
Antiplatelet or anticoagulant treatment	515 (3.3)	186 (2.8)	.04	433 (5.2)	211 (4.2)	.04	4.2 (0.0)	4.2 (0.0)	>.99

Abbreviations: BMI, body mass index (calculated as weight in kilograms divided by height in meters squared); HbA_1c_, hemoglobin A_1c_; HDL, high-density lipoprotein; IMD, Index of Multiple Deprivation; LDL, low-density lipoprotein.

SI conversion factors: To convert HbA_1c_ level to proportion of total hemoglobin, multiply by 0.01; to convert total, HDL, and LDL cholesterol level to milligrams per deciliter, divide by 0.0259; to convert triglyceride level to milligrams per deciliter, divide by 0.0113; and to convert creatinine level to milligrams per deciliter, divide by 88.4.

^a^ Data are presented as the number (percentage) of participants unless otherwise indicated.

**Table 2.** Characteristics in the Comparison Cohorts of New Zealand European and Pasifika Individuals With Type 2 Diabetes in New Zealand From 1994 to 2018^a^

Characteristic	Unmatched cohorts			Cohorts after coarsened exact matching			Cohorts after entropy matching		
	New Zealand European (n = 15 469)	Pasifika (n = 11 399)	*P* value	New Zealand European (n = 9340)	Pasifika (n = 8828)	*P* value	New Zealand European (n = 9340)	Pasifika (n = 8828)	*P* value
Age, mean (SD), y	61.6 (13.2)	52.8 (12.7)	<.001	60.6 (13.1)	53.1 (12.6)	<.001	51.9 (11.9)	51.9 (11.9)	>.99
Sex									
Female	6947 (44.9)	5994 (52.6)	<.001	4455 (47.7)	4612 (52.2)	<.001	50.7 (0.0)	50.7 (0.0)	>.99
Male	8522 (55.1)	5405 (47.4)	<.001	4885 (52.3)	4216 (47.8)	<.001	49.3 (0.0)	49.3 (0.0)	>.99
Enrollment cohort									
1994-1998	4716 (30.5)	2385 (20.9)	<.001	2965 (31.8)	1794 (20.3)	<.001	11.5 (0.0)	10.8 (0.0)	.17
1999-2003	4237 (27.4)	3087 (27.1)		2337 (25.0)	2404 (27.2)		16.2 (0.0)	18.9 (0.0)	
2004-2008	3054 (19.7)	1485 (13.0)		1919 (20.6)	1129 (12.8)		20.8 (0.0)	16.7 (0.0)	
2009-2013	2426 (15.7)	2882 (25.3)		1575 (16.9)	2354 (26.7)		34.5 (0.0)	37.1 (0.0)	
2013-2018	1036 (6.7)	1560 (13.7)		544 (5.8)	1147 (13.0)		17.1 (0.0)	16.5 (0.0)	
Duration of diabetes, mean (SD), y	3.9 (1.1)	4.2 (1.0)	<.001	4.1 (1.2)	4.2 (1.0)	.34	4.0 (0.8)	4.0 (0.8)	>.99
IMD group (NZDep2013 scale score)									
1 (1 or 2)	2650 (19.2)	288 (2.8)	<.001	1375 (14.7)	239 (2.7)	<.001	3.0 (0.0)	2.8 (0.0)	.39
2 (3 or 4)	2370 (17.1)	510 (4.9)		1263 (13.5)	433 (4.9)		4.4 (0.0)	5.3 (0.0)	
3 (5 or 6)	2162 (15.6)	561 (5.4)		1102 (11.8)	473 (5.4)		6.3 (0.0)	5.0 (0.0)	
4 (7 or 8)	3624 (26.2)	2189 (21.1)		3055 (32.7)	1871 (21.2)		20.6 (0.0)	21.5 (0.0)	
5 (9 or 10)	3018 (21.8)	6850 (65.9)		2545 (27.3)	5812 (65.8)		65.6 (0.0)	65.4 (0.0)	
Smoking status									
Never	10 543 (68.2)	8012 (70.3)	<.001	6851 (73.4)	6318 (71.6)	<.001	65.6 (0.0)	65.6 (0.0)	>.99
Former	3226 (20.9)	1740 (15.3)		1610 (17.2)	1323 (15.0)		19.1 (0.0)	19.1 (0.0)	
Current	1700 (11.0)	1647 (14.5)		879 (9.4)	1187 (13.5)		15.2 (0.0)	15.2 (0.0)	
BMI, mean (SD)	31.2 (6.4)	34.8 (7.2)	<.001	31.9 (6.5)	34.8 (7.2)	<.001	35.0 (7.2)	35.0 (7.2)	>.99
Blood pressure, mean (SD), mm Hg									
Systolic	138 (18)	132 (17)	<.001	139 (18)	133 (17)	<.001	132 (17)	132 (17)	>.99
Diastolic	80 (10)	82 (11)	<.001	80 (10)	82 (11)	<.001	81 (11)	81 (11)	>.99
HbA_1c_ level, mean (SD), mmol/mol	54.2 (17.3)	67.3 (21.4)	<.001	56.3 (17.1)	67.6 (21.5)	<.001	66.0 (20.5)	66.0 (20.5)	>.99
Total cholesterol level, mean (SD), mmol/L	5.1 (1.2)	5.0 (1.2)	<.001	5.1 (1.2)	5.1 (1.2)	.003	4.9 (1.2)	4.9 (1.2)	>.99
Triglyceride level, mean (SD), mmol/L	2.2 (1.5)	2.2 (1.7)	<.001	2.2 (1.4)	2.2 (1.7)	.003	2.0 (1.3)	2.0 (1.3)	>.99
LDL cholesterol level, mean (SD), mmol/L	2.5 (0.9)	2.6 (1.0)	<.001	2.6 (0.9)	2.6 (1.0)	.002	2.6 (1.0)	2.6 (1.0)	>.99
HDL cholesterol level, mean (SD), mmol/L	1.2 (0.4)	1.2 (0.3)	<.001	1.2 (0.4)	1.2 (0.3)	.003	1.2 (0.3)	1.2 (0.3)	>.99
Creatinine level, mean (SD), μmol/L	85.5 (18.4)	81.8 (20.1)	<.001	82.0 (18.6)	81.6 (20.4)	.004	79.9 (19.7)	79.9 (19.7)	>.99
Antidiabetes treatment									
Oral drug and insulin	2398 (15.5)	2069 (18.2)	<.001	1841 (19.7)	1785 (20.2)	<.001	22.9 (0.0)	24.0 (0.0)	.27
Oral drug only	8640 (55.9)	7174 (62.9)		5787 (62.0)	5737 (65.0)		70.1 (0.0)	69.0 (0.0)	
Insulin only	571 (3.7)	286 (2.5)		351 (3.8)	176 (2.0)		2.0 (0.0)	0.8 (0.0)	
Antihypertensive treatment	10 676 (69.0)	7604 (66.7)	<.001	6988 (74.8)	6420 (72.7)	.001	84.0 (0.0)	84.0 (0.0)	>.99
Statin treatment	8556 (55.3)	5986 (52.5)	<.001	7220 (77.3)	6921 (78.4)	.003	78.4 (0.0)	78.4 (0.0)	>.99
Antiplatelet or anticoagulant treatment	515 (3.3)	168 (1.5)	<.001	224 (2.4)	177 (2.0)	.001	2.3 (0.0)	2.3 (0.0)	>.99

Abbreviations: BMI, body mass index (calculated as weight in kilograms divided by height in meters squared); HbA_1c_, hemoglobin A_1c_; HDL, high-density lipoprotein; IMD, Index of Multiple Deprivation; LDL, low-density lipoprotein.

SI conversion factors: To convert HbA_1c_ level to proportion of total hemoglobin, multiply by 0.01; to convert total, HDL, and LDL cholesterol level to milligrams per deciliter, divide by 0.0259; to convert triglyceride level to milligrams per deciliter, divide by 0.0113; and to convert creatinine level to milligrams per deciliter, divide by 88.4.

^a^ Data are presented as the number (percentage) of participants unless otherwise indicated.

### Rates of Site-Specific Cancers in New Zealand European vs Māori Individuals With Type 2 Diabetes

**Figure 1.**
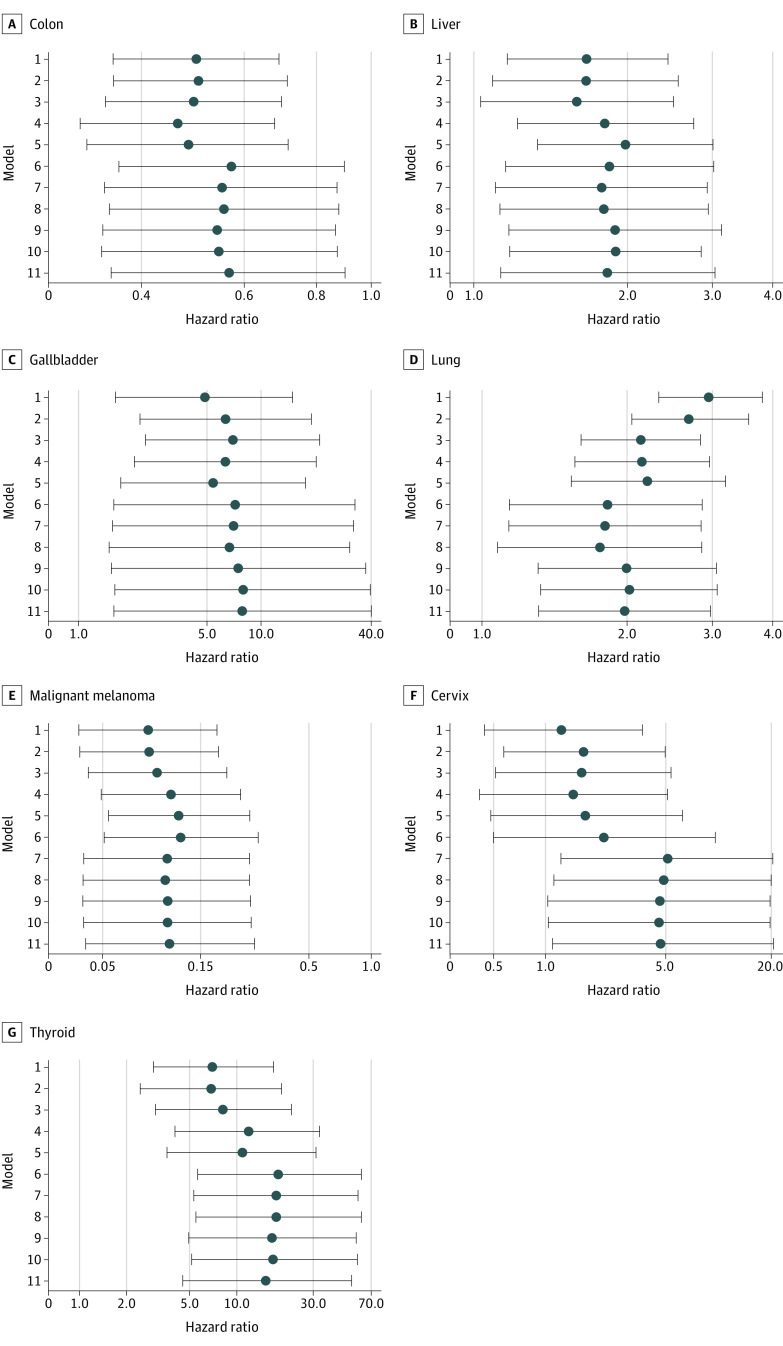
Adjusted Hazard Ratios for the Association Between Māori Ethnicity vs New Zealand European Ethnicity and Risk of 7 Site-Specific Cancers Whiskers represent 95% CIs.

The rates of each site-specific cancer in the unmatched and matched cohorts between New Zealand European and Māori individuals with type 2 diabetes and between New Zealand European and Pasifika individuals with type 2 diabetes are presented in Table 3. Compared with New Zealand European individuals with type 2 diabetes, Māori individuals with type 2 diabetes had a decreased risk of colon cancer (HR, 0.56; 95% CI, 0.35-0.90) and malignant melanoma (HR, 0.11; 95% CI, 0.04-0.27); however, they had an increased risk of liver cancer (HR, 1.81; 95% CI, 1.08-3.03), gallbladder cancer (HR, 7.94; 95% CI, 1.57-40.24), lung cancer (HR, 1.97; 95% CI, 1.30-2.99), and thyroid cancer (HR, 15.36; 95% CI, 4.50-52.34) based on model 11 (Figure 1 and eTable 3 in the Supplement). Among women, compared with New Zealand European women with type 2 diabetes, Māori women with type 2 diabetes had an increased risk of cervical cancer (HR, 4.81; 95% CI, 1.08-21.42) based on model 11. Figure 1 and eFigure 10 and eTable 3 in the Supplement show the HRs (95% CIs) with each matching step for each of the 21 cancers.

**Table 3.** Distribution of Cancer Outcomes in the Comparative Cohorts

Cancer site	Māori vs New Zealand European, No. (%)				Pasifika vs New Zealand European, No. (%)			
	Unmatched cohorts		CEM matched cohorts^a^		Unmatched cohorts		CEM matched cohorts^a^	
	New Zealand European	Māori	New Zealand European	Māori	New Zealand European	Pasifika	New Zealand European	Pasifika
**Overall**								
Participants, No.	15 469	6656	8361	5039	15 469	11 399	9340	8828
Oral cavity	42 (0.3)	12 (0.2)	19 (0.2)	8 (0.2)	42 (0.3)	14 (0.1)	19 (0.2)	10 (0.1)
Esophagus	59 (0.4)	8 (0.1)	38 (0.5)	5 (0.1)	59 (0.4)	8 (0.1)	34 (0.4)	7 (0.1)
Stomach	76 (0.5)	40 (0.6)	43 (0.5)	30 (0.6)	76 (0.5)	69 (0.6)	50 (0.5)	56 (0.6)
Colon	360 (2.3)	66 (1.0)	209 (2.5)	45 (0.9)	360 (2.3)	73 (0.6)	232 (2.5)	57 (0.7)
Rectum	121 (0.8)	30 (0.5)	75 (0.9)	24 (0.5)	121 (0.8)	30 (0.3)	83 (0.9)	25 (0.3)
Liver	135 (0.9)	76 (1.1)	72 (0.9)	57 (1.1)	136 (0.9)	71 (0.6)	79 (0.9)	57 (0.7)
Gallbladder	9 (0.1)	12 (0.2)	5 (0.1)	11 (0.2)	9 (0.1)	10 (0.1)	4 (0.04)	9 (0.1)
Pancreas	119 (0.8)	32 (0.5)	68 (0.8)	31 (0.6)	119 (0.8)	32 (0.3)	78 (0.8)	27 (0.3)
Lung	198 (1.9)	240 (3.6)	146 (1.8)	176 (3.5)	298 (1.9)	132 (1.2)	151 (1.6)	94 (1.1)
Malignant melanoma	277 (1.8)	8 (0.1)	167 (2.0)	7 (0.1)	277 (1.8)	8 (0.1)	191 (2.0)	4 (1.0)
Kidney	86 (0.6)	40 (0.6)	45 (0.5)	27 (0.5)	86 (0.6)	28 (0.3)	54 (0.6)	18 (0.2)
Bladder	147 (1.0)	14 (0.2)	87 (1.0)	13 (0.3)	147 (1.0)	13 (0.1)	81 (0.9)	8 (0.1)
Brain and CNS	32 (0.2)	9 (0.1)	15 (0.2)	7 (0.1)	32 (0.2)	11 (0.1)	13 (0.1)	8 (0.1)
Thyroid	15 (0.1)	28 (0.4)	7 (0.1)	21 (0.4)	15 (0.1)	14 (0.1)	10 (0.1)	11 (0.1)
Non-Hodgkin lymphoma	109 (0.7)	35 (0.5)	67 (0.8)	23 (0.5)	109 (0.7)	37 (0.3)	69 (0.7)	29 (0.3)
Multiple myeloma	38 (0.3)	13 (0.2)	18 (0.2)	9 (0.2)	38 (0.3)	25 (0.2)	27 (0.3)	18 (0.2)
Leukemia	108 (0.7)	40 (0.6)	54 (0.7)	30 (0.6)	100 (0.7)	32 (0.3)	57 (0.6)	22 (0.3)
**Female**								
Participants, No.	6940	3341	3766	2488	6940	5984	4455	4612
Breast	269 (3.9)	130 (3.9)	165 (4.4)	101 (4.1)	269 (3.9)	117 (2.0)	191 (14.3)	94 (2.0)
Cervix	12 (0.2)	11 (0.3)	8 (0.21)	7 (0.28)	12 (0.2)	15 (0.3)	8 (0.2)	15 (0.3)
Ovarian	41 (0.6)	12 (0.4)	24 (0.6)	10 (0.4)	41 (0.6)	28 (0.5)	28 (0.6)	25 (0.5)
**Male**								
Participants, No.	8522	3311	4595	2542	8522	5405	4885	4216
Prostate	416 (4.9)	83 (2.5)	228 (5.0)	74 (2.9)	416 (4.9)	117 (2.2)	243 (5.0)	91 (2.2)

Abbreviations: CEM, coarsened exact matching; CNS, central nervous system.

^a^ The sample size of cohorts after CEM was the same as those in entropy balancing.

### Rates of Site-Specific Cancers in New Zealand European vs Pasifika Individuals With Type 2 Diabetes

**Figure 2.**
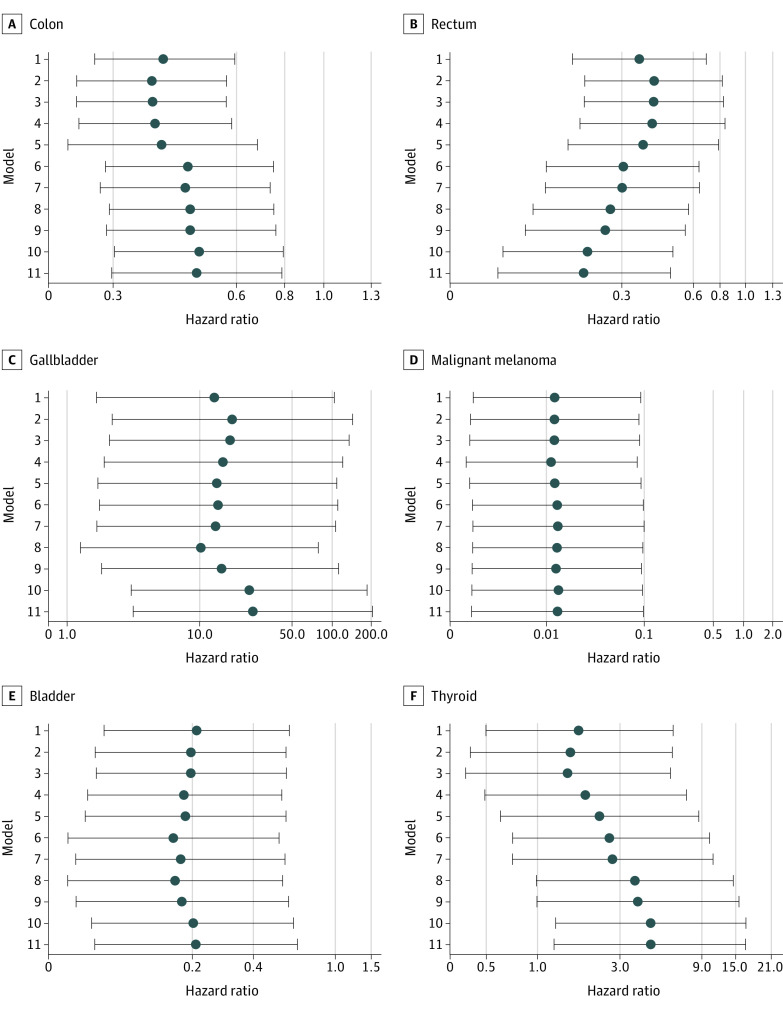
Adjusted Hazard Ratios for the Association Between Pasifika Ethnicity vs New Zealand European Ethnicity and Risk of 6 Site-Specific Cancers Whiskers represent 95% CIs.

Compared with New Zealand European individuals with type 2 diabetes, Pasifika individuals with type 2 diabetes had an overall decreased risk of colon cancer (HR, 0.48; 95% CI, 0.30-0.78), rectal cancer (HR, 0.21; 95% CI, 0.09-0.48), malignant melanoma (HR, 0.21; 95% CI, 0.07-0.65), and bladder cancer (HR, 0.01; 95% CI, 0.01-0.10) and an increased risk of gallbladder cancer (HR, 25.10; 95% CI, 3.14-200.63) and thyroid cancer (HR, 4.47; 95% CI, 1.25-16.03) (Figure 2 and eTable 4 in the Supplement). Figure 2 and eFigure 11 and eTable 4 in the Supplement show the HRs (95% CIs) with each matching step for each of the 21 cancers.

## Discussion

Few studies have examined ethnic differences in cancer risk among patients with type 2 diabetes. We compared the incidence of 21 adult cancers for a 25-year period (1994-2018) between New Zealand European and Māori individuals and between New Zealand European and Pasifika individuals with type 2 diabetes. After matching for demographic characteristics, socioeconomic status, lifestyle factors, routine clinical measurements, treatments, period effects, and duration of diabetes, cancer risk among ethnic groups varied substantially by cancer type in both direction and size. Compared with New Zealand European individuals, Māori individuals were more likely to have an increased risk of liver, gallbladder, lung, and thyroid cancers and a decreased risk of colon cancer and malignant melanoma, whereas Pasifika individuals were more likely to have an increased risk of gallbladder and thyroid cancers and a decreased risk of colon cancer, rectal cancer, malignant melanoma, and bladder cancer. Compared with New Zealand European women, Māori women also had a higher risk of cervical cancer.

These findings are consistent in part with New Zealand Ministry of Health data, which report that, nationally, Māori individuals have a higher incidence of potentially preventable cancers than do non-Māori individuals (most of whom are of European descent).^20,21^ From 2010 to 2012, rates of breast, lung, uterine, and cervical cancers among Māori women were significantly higher than those among non-Māori women except for colorectal cancer, for which the rate was lower.^2^ Rates of lung, liver, and stomach cancers were higher among Māori men than among non-Māori men, whereas prostate and colorectal cancer rates were lower.^20,21^ Age-standardized rates of cancers of the cervix, endometrium, gallbladder, lip, mouth and pharynx, liver, lung, ovary, pancreas, stomach, and thyroid are higher among Pasifika individuals living in New Zealand compared with the general population, whereas rates of colorectal, bladder, and testicular cancers and melanoma are lower.^10^

The lower risk of colorectal cancer persists despite the high prevalence of factors associated with colorectal cancer among the Pasifika and Māori populations compared with the general New Zealand population and the increase in colorectal cancer rates among Māori individuals over time.^21^ The risk of colorectal cancer in the Māori and Pasifika populations was even lower among the sample of Māori and Pasifika individuals with type 2 diabetes in the present study. However, our results contradict previous findings of an increased risk of colorectal cancer among individuals with type 2 diabetes compared with populations without diabetes.^22,23^ Further studies are needed to explore factors that potentially protect against colorectal cancer among the New Zealand Pasifika and Māori populations, including the role of widespread metformin use and possible dietary factors.^23^

In the present study, a higher risk of lung cancer was observed in Māori individuals than in New Zealand European individuals with type 2 diabetes. These results are consistent with previous findings in the general New Zealand population, in which Māori individuals consistently have had a higher risk of lung cancer.^24^ In contrast, although Pasifika individuals have a higher risk of lung cancer compared with New Zealand European individuals,^22^ consistent with higher smoking rates,^10^ in the cohort with type 2 diabetes in the present study, Pasifika individuals had a lower risk of lung cancer than did New Zealand European individuals.

Among individuals with type 2 diabetes, the risk of liver cancer was higher among Māori individuals but not among Pasifika individuals compared with New Zealand European individuals. This finding differs from that in the general population, in which both the Māori and the Pasifika communities are at increased risk.^2,24^ Hepatitis B is the main factor associated with liver cancer,^25^ and among Māori individuals, hepatitis B surface antigen carriage was previously found to explain most of the excess standardized rate of liver cancer compared with the rate among New Zealand European individuals.^26^ Information on hepatitis B surface antigen carriage and hepatitis B immunization was unavailable in this study. The increased risk for cervical cancer and reduced risk for malignant melanoma among both Māori and Pasifika individuals with type 2 diabetes were also consistent with findings in the general population.^22^

Although in the present study, an increased risk for gallbladder cancer was found in both Māori and Pasifika individuals with type 2 diabetes compared with New Zealand European individuals with type 2 diabetes, consistent with findings from the general population,^10,27^ absolute numbers were small. Information on gallstone incidence, the predominant factor associated with gallbladder cancer,^28^ was unavailable in the present study.^29^ Increased surveillance for gallstones may be warranted among Pasifika and Māori individuals with type 2 diabetes given the increased risk of gallbladder cancer found in these groups in the present study.

In the general New Zealand population, Māori and Pasifika individuals are reported to be approximately one-third more likely and twice as likely, respectively, than non-Māori individuals to be diagnosed with thyroid cancer.^21,29^ This differential risk was also found in the current study, in which thyroid cancer risk was greater among Māori and Pasifika individuals compared with New Zealand European individuals. Although we matched sex and obesity status, data on other factors associated with thyroid cancer (eg, diet, family history of cancer, and thyroid disease)^29^ were unavailable.

Unlike in the general New Zealand population, in the population with type 2 diabetes in the present study,^6^ only Pasifika, not Māori, individuals had a lower risk of bladder cancer compared with New Zealand European individuals. Although we matched for some factors associated with bladder cancer, including age, sex, smoking status, and use of diabetes medications, data on other important factors associated with bladder cancer (eg, chronic bladder infections)^30^ were unavailable.

### Strengths and Limitations

This study has strengths. To our knowledge, this was the largest study of multiethnic matched cohorts of participants with type 2 diabetes in New Zealand to report the incidence of 21 common site-specific cancers during a 25-year period. These cohorts included all patients from participating general practices. By linking large, nationally representative databases, we were able to obtain prospective follow-up data on patients to ascertain all incident site-specific cancers. All cancers included in this study were based on the linkage of specific registration data sets that had good validation of outcomes. The accuracy of clinical recording and diagnoses in this study was validated for outcomes defined by *ICD-9* and *ICD-10* codes, which have high precision.^31^ Another strength of this study was the application of a novel, tapered matching method to form comparison cohorts to assess the risk for cancers among patients with type 2 diabetes from different ethnic groups. Through tapered matching, we were able to examine whether differences in specific sets of confounders were associated with the risk for cancers. By sequentially controlling for differences in demographic characteristics, socioeconomic status, lifestyle factors, body measurements, key clinical measurements routinely provided to patients with type 2 diabetes, antidiabetes treatments, antihypertensive treatments, anticoagulant treatments, period effects of enrollment, and duration of diabetes, we could evaluate how the risks for cancers compared after each match between patients from different ethnic groups.

This study also has limitations. These included the heterogeneity in the Pasifika, Māori, and New Zealand European cohorts and the national representativeness of the sample and of the participating general practices. In addition, information on certain factors associated with some of the cancers examined in this study was not available, including cumulative exposure of tobacco use, dietary information, prevalence of gallstones, hepatitis B and human papillomavirus serologic test results, and exposure to industrial chemicals. Future replication studies taking these factors into account are warranted. Although both CEM and entropy balancing were performed without reference to outcomes, the sample size and the weights remained the same for each outcome in the entropy balancing. Because CEM involves excluding some participants (depending on the coarsening), there was potentially a change in the estimate if the outcome was rare (eg, oral cavity or esophageal cancer); the HRs identified for such rare outcomes need to be further validated in external studies with larger sample sizes.

## Conclusions

In this cohort study of adults with type 2 diabetes in New Zealand from 1994 to 2018, after matching for possible confounders, significantly higher risks for liver, gallbladder, lung, and thyroid cancers were found among Māori and Pasifika individuals compared with New Zealand European individuals and higher rates of malignant melanoma and colorectal cancer were found among New Zealand European individuals. These data suggest a need to consider specific cancer prevention and detection activities beyond diabetes lifestyle management and improved cancer screening among people with type 2 diabetes. Research into the biological, health service, and social mechanisms underlying differences in cancer risk among ethnic groups appears to be needed.

## Notes

Supplement. **eFigure 1.** Workflow Charts for Matching Process (NZ European vs Māori) **eFigure 2.** Workflow Charts for Matching Process (NZ European vs Pasifika) **eFigure 3.** Workflow Charts for Matching Process (NZ European Female vs Māori Female) **eFigure 4.** Workflow Charts for Matching Process (NZ European Female vs Pasifika Female) **eFigure 5**. Workflow Charts for Matching Process (NZ European Male vs Māori Male) **eFigure 6.** Workflow Charts for Matching Process (NZ European Male vs Pasifika Male) **eFigure 7.** Distribution of Difference of Means, Variance, and Skewness on Matched Variables in the Unmatched and Matched Cohorts **eFigure 8.** Distribution of Difference of Means, Variance, and Skewness on Matched Variables in the Unmatched and Matched Cohorts in Females **eFigure 9.** Distribution of Difference of Means, Variance, and Skewness on Matched Variables in the Unmatched and Matched Cohorts in Males **eFigure 10.** Adjusted Hazard Ratios for Association Between Māori (Reference to NZ European) and Risks of 14 Cancers **eFigure 11.** Adjusted Hazard Ratios for Association Between Pasifika (Reference to NZ European) and Risks of 15 Cancers **eTable 1.** Characteristics in the Comparison Female Cohorts **eTable 2.** Characteristics in the Comparison Male Cohorts **eTable 3.** Adjusted Hazard Ratios for Association Between Māori (Reference to NZ European) and Risks of 21 Cancers **eTable 4.** Adjusted Hazard Ratios for Association Between Pasifika (Reference to NZ European) and Risks of 21 Cancers
