# Supplementary material for: Ethnic Differences in Cancer Rates Among Adults With Type 2 Diabetes in New Zealand From 1994 to 2018
Source: JAMA Netw Open. 2022 Feb 7;5(2):e2147171. doi: 10.1001/jamanetworkopen.2021.47171 (PMC8822383; doi:10.1001/jamanetworkopen.2021.47171)
Supplement: Supplement. — eFigure 1. Workflow Charts for Matching Process (NZ European vs Māori) eFigure 2. Workflow Charts for Matching Process (NZ European vs Pasifika) eFigure 3. Workflow Charts for Matching Process (NZ European Female vs Māori Female) eFigure 4. Workflow Charts for Matching Process (NZ European Female vs Pasifika Female) eFigure 5. Workflow Charts for Matching Process (NZ European Male vs Māori Male) eFigure 6. Workflow Charts for Matching Process (NZ European Male vs Pasifika Male) eFigure 7. Distribution of Difference of Means, Variance, and Skewness on Matched Variables in the Unmatched and Matched Cohorts eFigure 8. Distribution of Difference of Means, Variance, and Skewness on Matched Variables in the Unmatched and Matched Cohorts in Females eFigure 9. Distribution of Difference of Means, Variance, and Skewness on Matched Variables in the Unmatched and Matched Cohorts in Males eFigure 10. Adjusted Hazard Ratios for Association Between Māori (Reference to NZ European) and Risks of 14 Cancers eFigure 11. Adjusted Hazard Ratios for Association Between Pasifika (Reference to NZ European) and Risks of 15 Cancers eTable 1. Characteristics in the Comparison Female Cohorts eTable 2. Characteristics in the Comparison Male Cohorts eTable 3. Adjusted Hazard Ratios for Association Between Māori (Reference to NZ European) and Risks of 21 Cancers eTable 4. Adjusted Hazard Ratios for Association Between Pasifika (Reference to NZ European) and Risks of 21 Cancers [file jamanetwopen-e2147171-s001.pdf]

Supplementary Online Content

Yu D, Wang Z, Cai Y, et al. Ethnic differences in cancer rates among adults with type 2 diabetes in New Zealand from 1994 to 2018. *JAMA Netw Open*. 2022;5(2):e2147171. doi:10.1001/jamanetworkopen.2021.47171

eFigure 1. Workflow Charts for Matching Process (NZ European vs Māori)
eFigure 2. Workflow Charts for Matching Process (NZ European vs Pasifika)
eFigure 3. Workflow Charts for Matching Process (NZ European Female vs Māori Female)
eFigure 4. Workflow Charts for Matching Process (NZ European Female vs Pasifika Female)
eFigure 5. Workflow Charts for Matching Process (NZ European Male vs Māori Male)
eFigure 6. Workflow Charts for Matching Process (NZ European Male vs Pasifika Male)
eFigure 7. Distribution of Difference of Means, Variance, and Skewness on Matched Variables in the Unmatched and Matched Cohorts
eFigure 8. Distribution of Difference of Means, Variance, and Skewness on Matched Variables in the Unmatched and Matched Cohorts in Females
eFigure 9. Distribution of Difference of Means, Variance, and Skewness on Matched Variables in the Unmatched and Matched Cohorts in Males
eFigure 10. Adjusted Hazard Ratios for Association Between Māori (Reference to NZ European) and Risks of 14 Cancers
eFigure 11. Adjusted Hazard Ratios for Association Between Pasifika (Reference to NZ European) and Risks of 15 Cancers
eTable 1. Characteristics in the Comparison Female Cohorts
eTable 2. Characteristics in the Comparison Male Cohorts
eTable 3. Adjusted Hazard Ratios for Association Between Māori (Reference to NZ European) and Risks of 21 Cancers
eTable 4. Adjusted Hazard Ratios for Association Between Pasifika (Reference to NZ European) and Risks of 21 Cancers

This supplementary material has been provided by the authors to give readers additional information about their work.

© 2022 Yu D et al. *JAMA Network Open*.

Online-only Supplemental Material

eFigure 1. Workflow charts for matching process (NZ European vs. Māori)

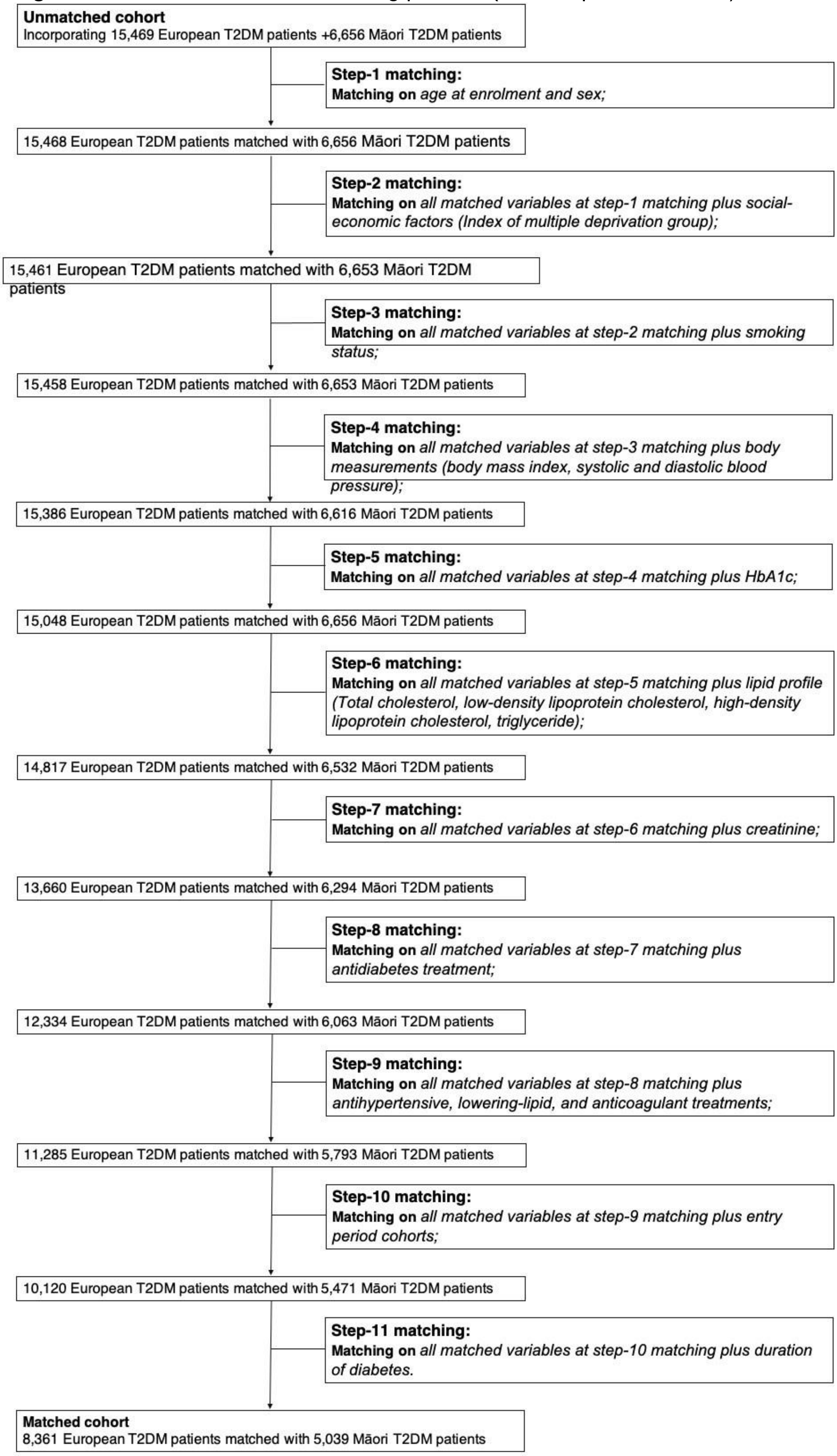

© 2022 Yu D et al. *JAMA Network Open*.

eFigure 2. Workflow charts for matching process (NZ European vs. Pasifika)

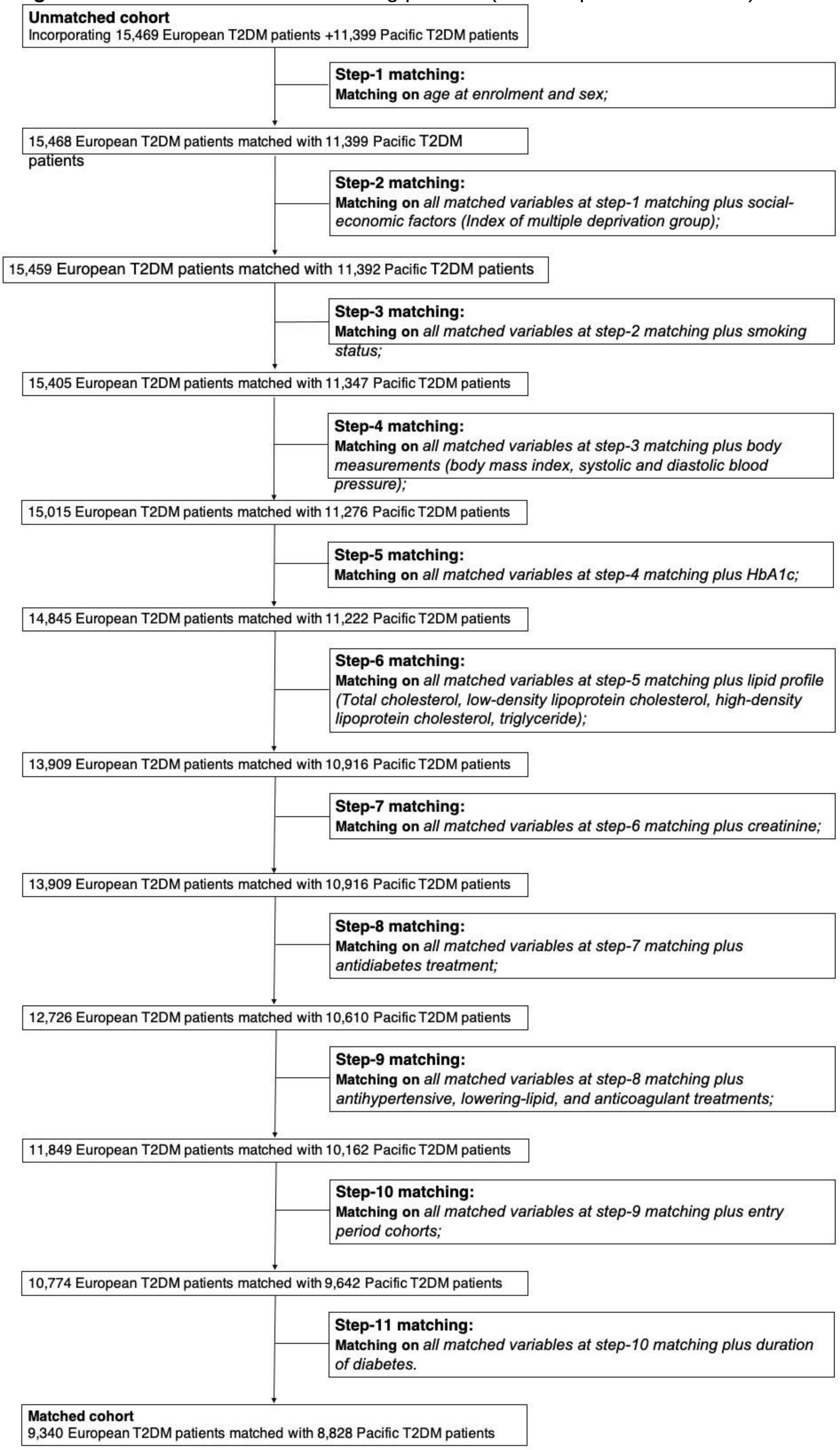

© 2022 Yu D et al. *JAMA Network Open*.

eFigure 3. Workflow charts for matching process (NZ European Female vs. Māori Female)

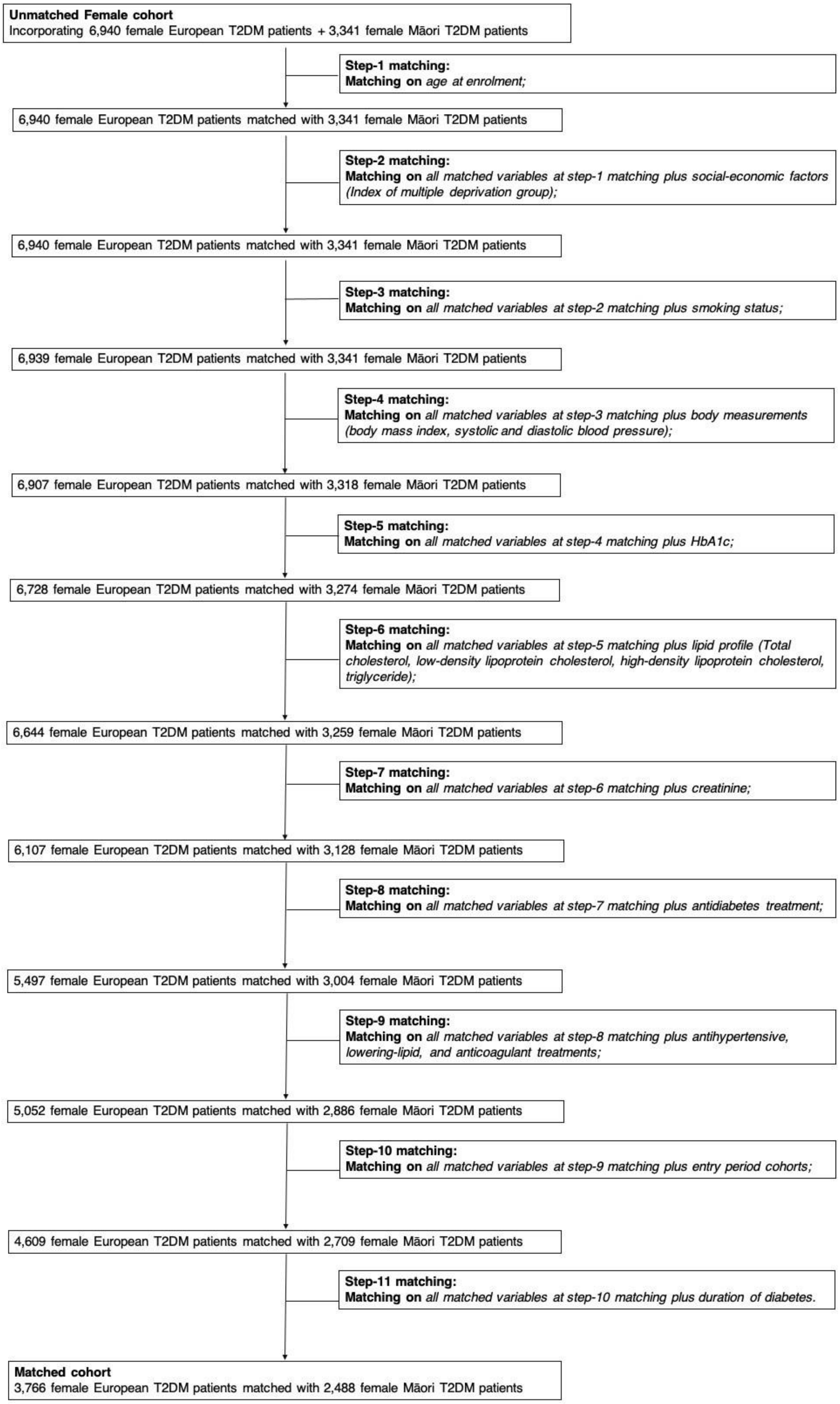

© 2022 Yu D et al. *JAMA Network Open*.

eFigure 4. Workflow charts for matching process (NZ European Female vs. Pasifika Female)

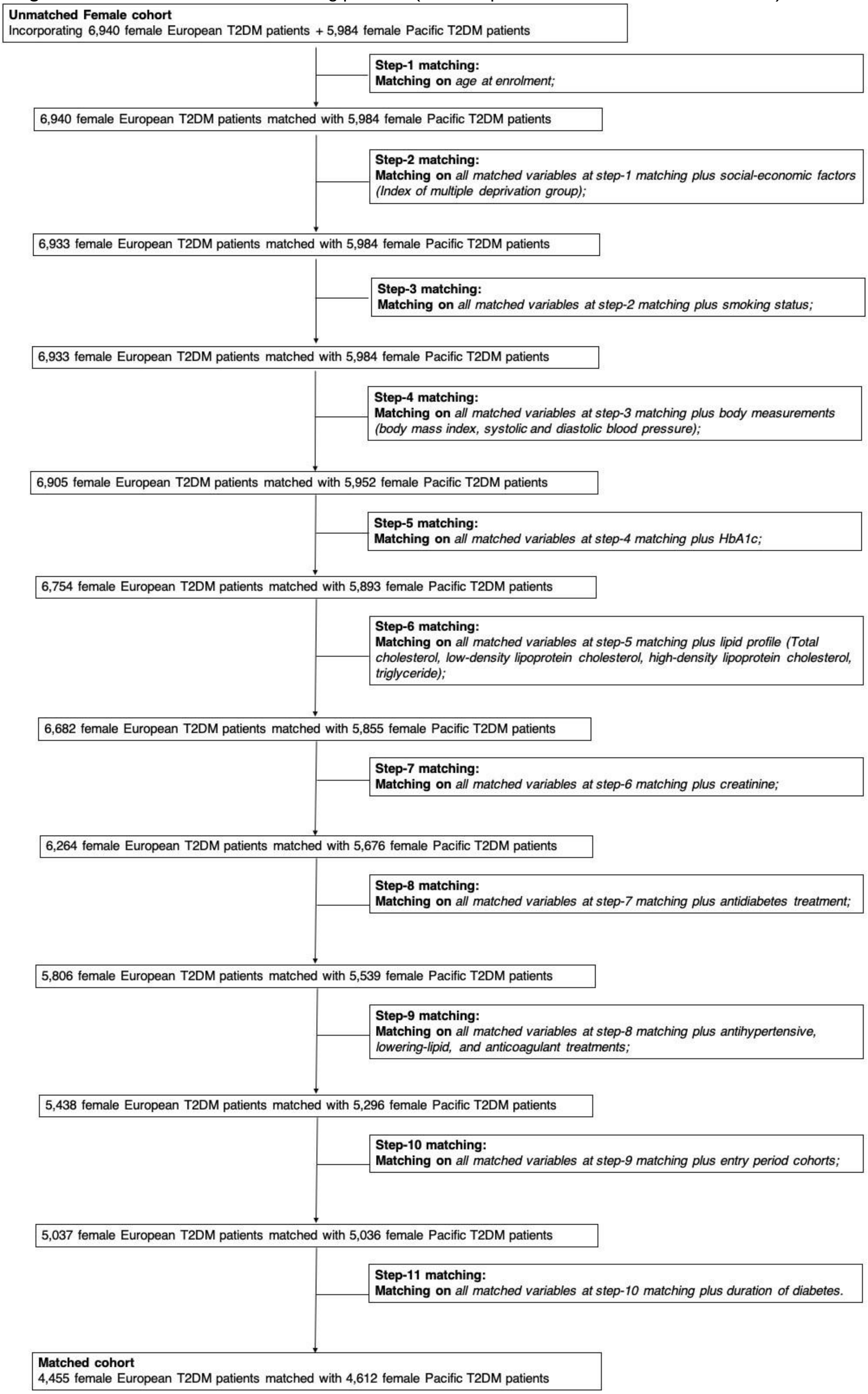

© 2022 Yu D et al. *JAMA Network Open*.

eFigure 5. Workflow charts for matching process (NZ European Male vs. Māori Male)

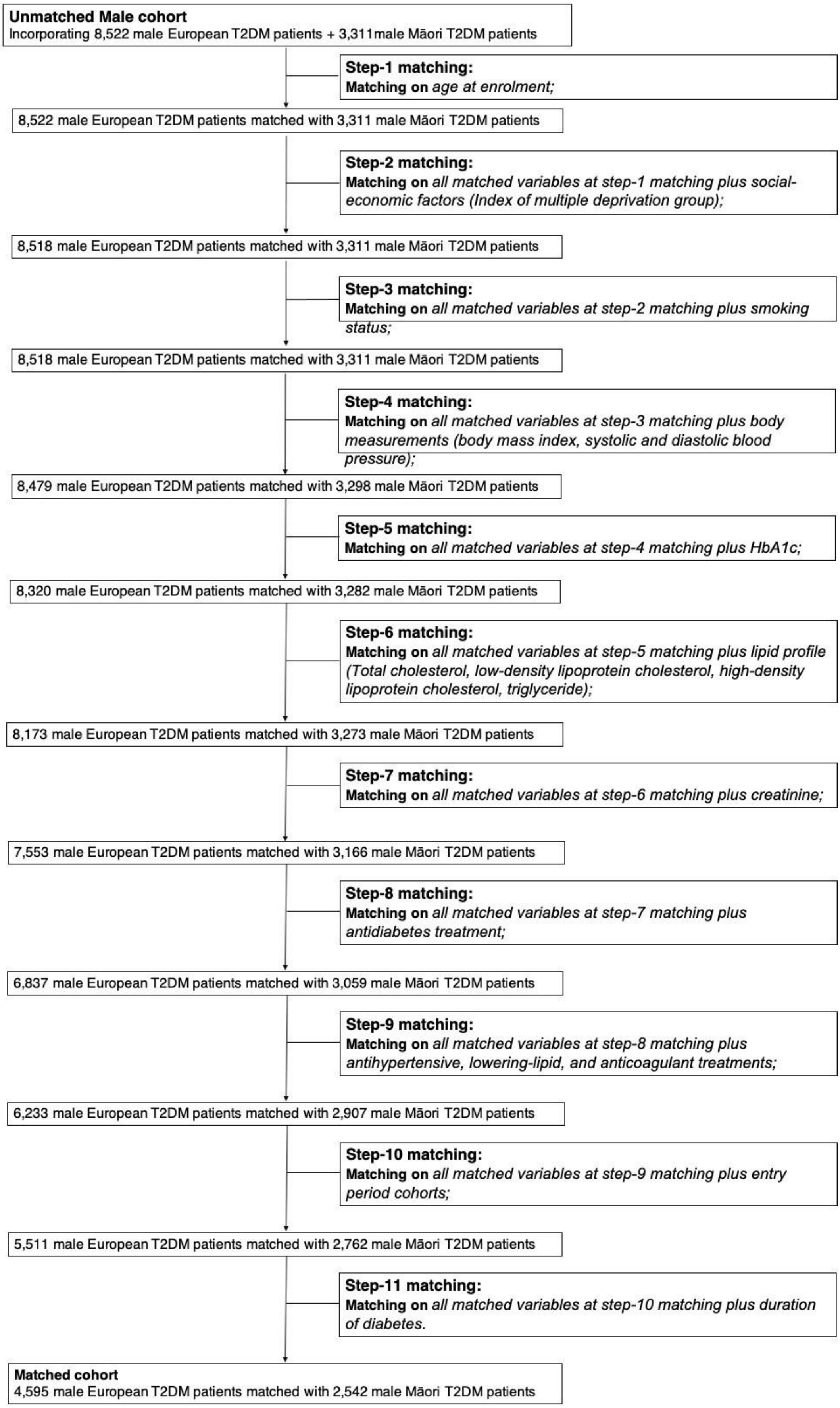

© 2022 Yu D et al. *JAMA Network Open*.

eFigure 6. Workflow charts for matching process (NZ European Male vs. Pasifika Male)

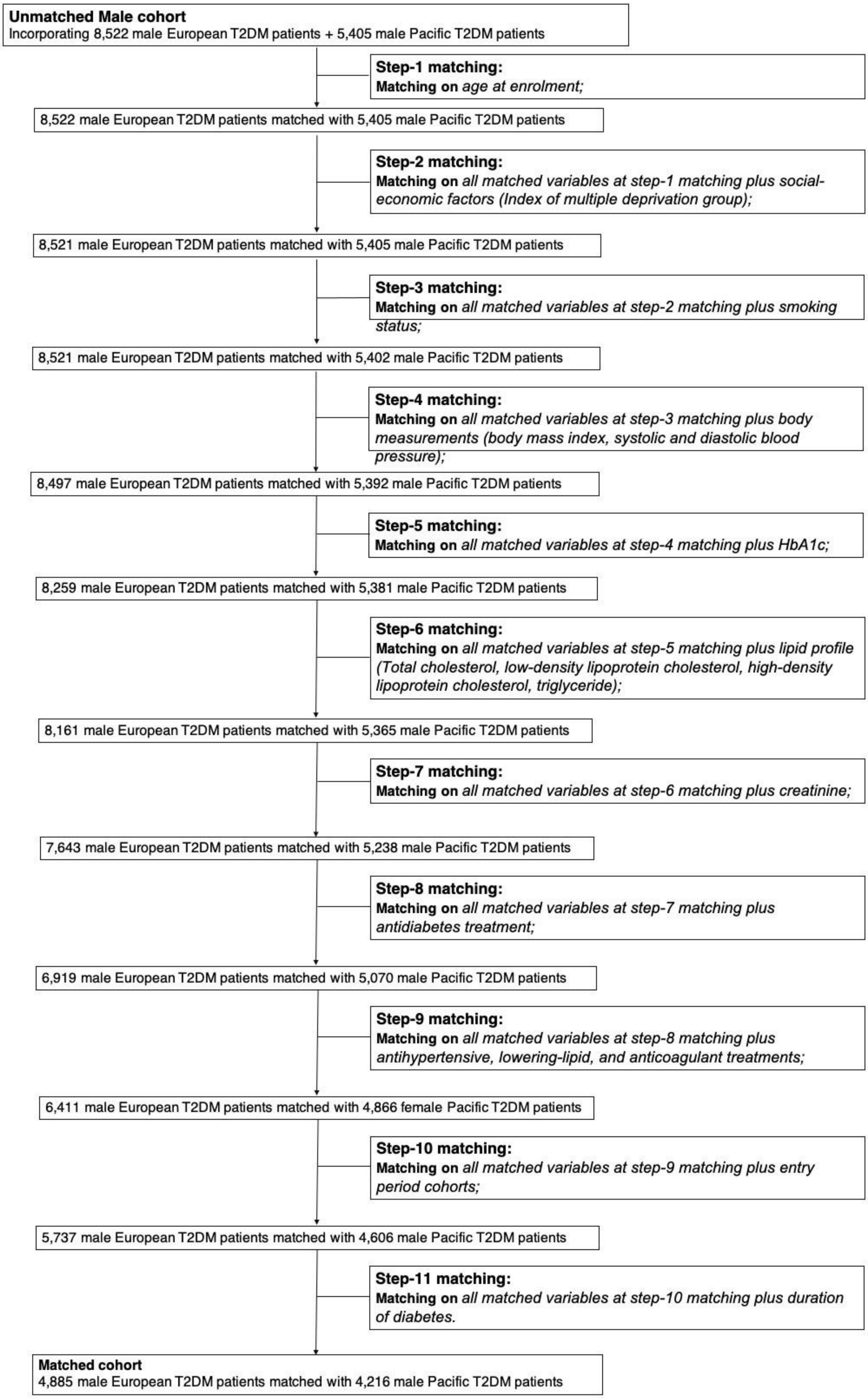

© 2022 Yu D et al. *JAMA Network Open*.

eFigure 7. Distribution of difference of means, variance and skewness on matched variables in the unmatched and matched cohorts.

Triangles indicate measurements from unmatched cohorts; diamonds indicate measurements from coarsened exact matching; circles indicate measurements from entropy matching cohorts

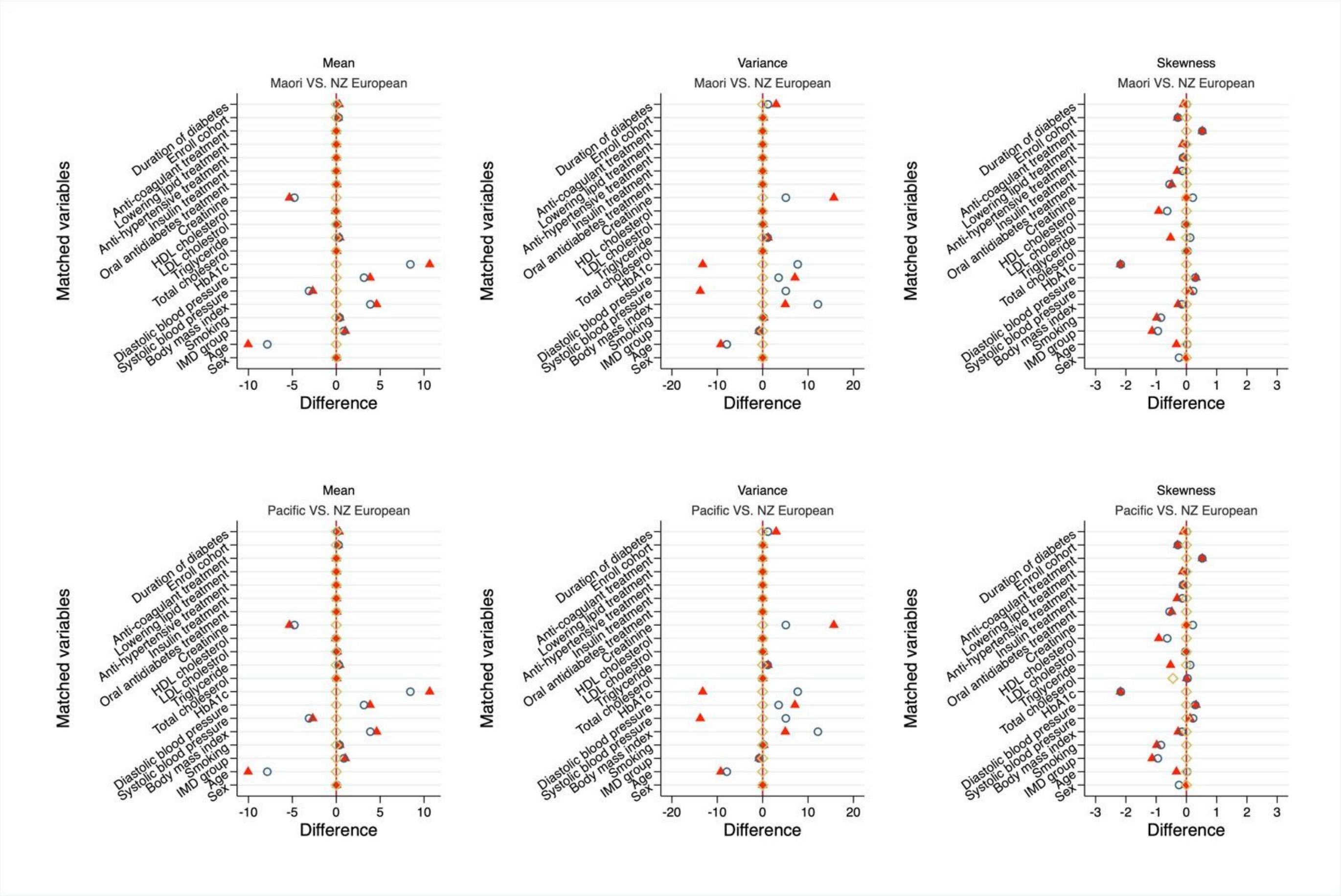

© 2022 Yu D et al. *JAMA Network Open*.

eFigure 8. Distribution of difference of means, variance and skewness on matched variables in the unmatched and matched cohorts in females

Triangles indicate measurements from unmatched cohorts; diamonds indicate measurements from coarsened exact matching; circles indicate measurements from entropy matching cohorts

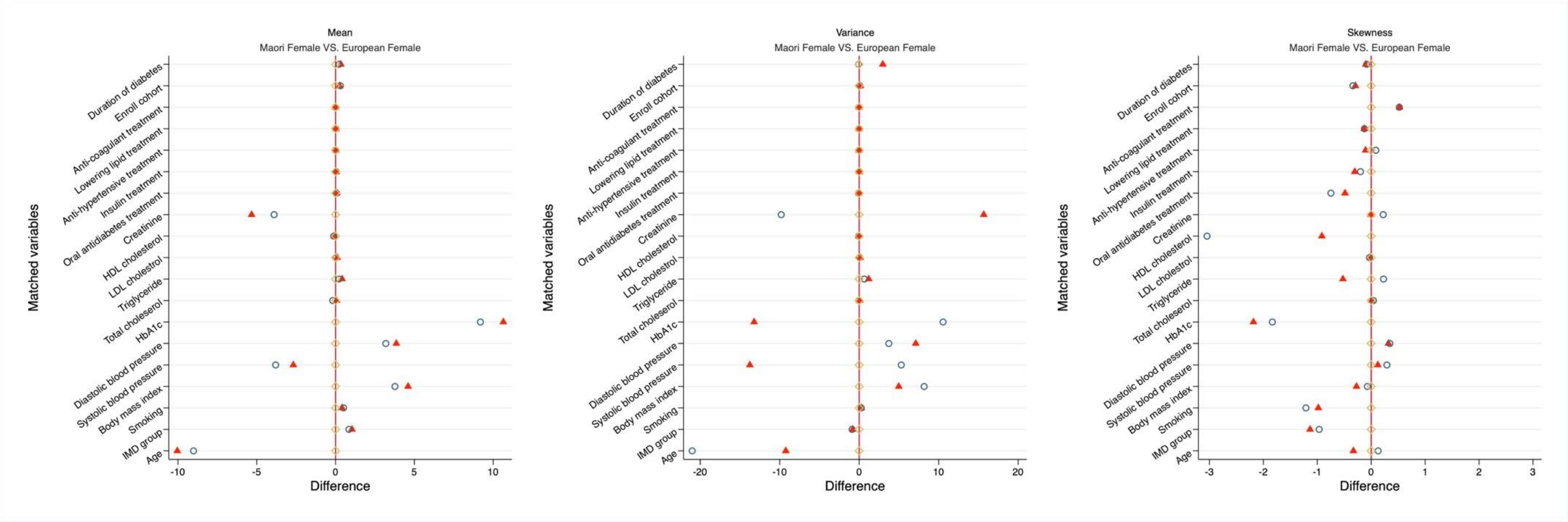

© 2022 Yu D et al. *JAMA Network Open*.

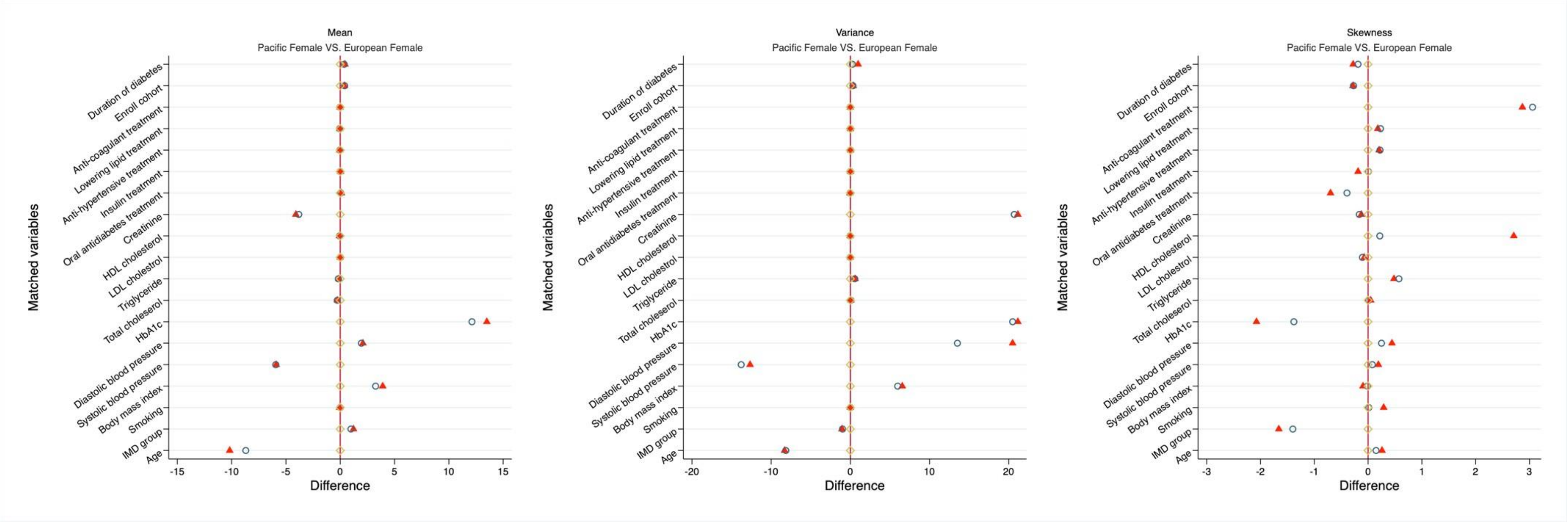

© 2022 Yu D et al. *JAMA Network Open*.

eFigure 9. Distribution of difference of means, variance and skewness on matched variables in the unmatched and matched cohorts in males

Triangles indicate measurements from unmatched cohorts; diamonds indicate measurements from coarsened exact matching; circles indicate measurements from entropy matching cohorts

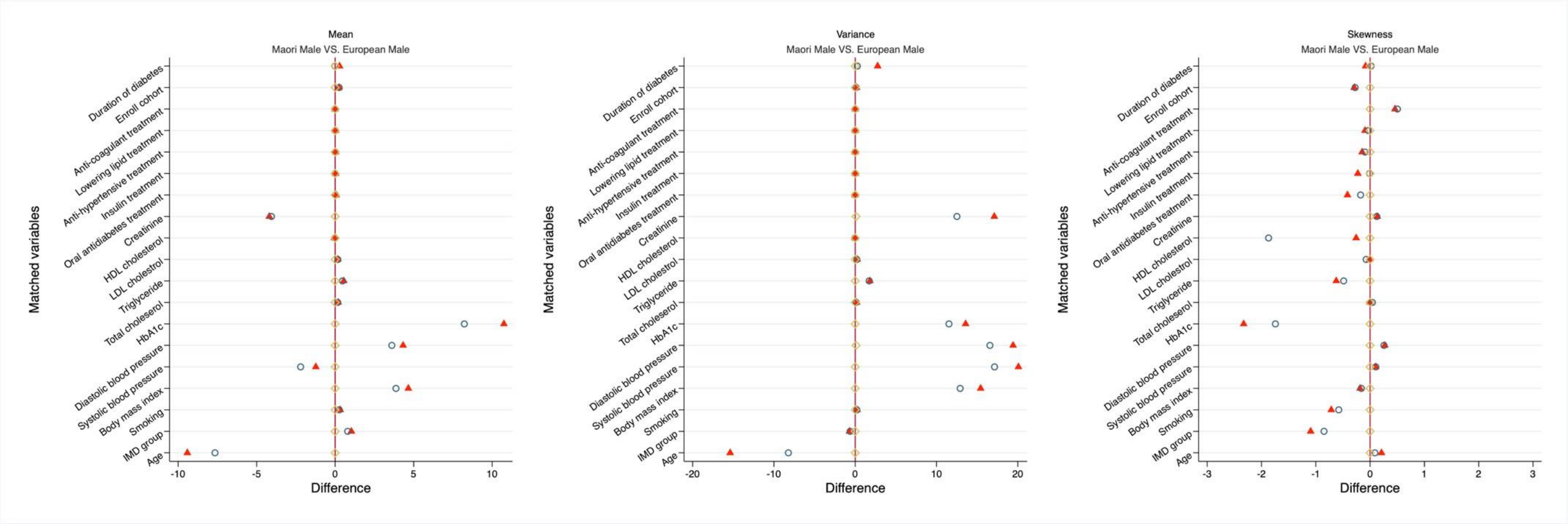

© 2022 Yu D et al. *JAMA Network Open*.

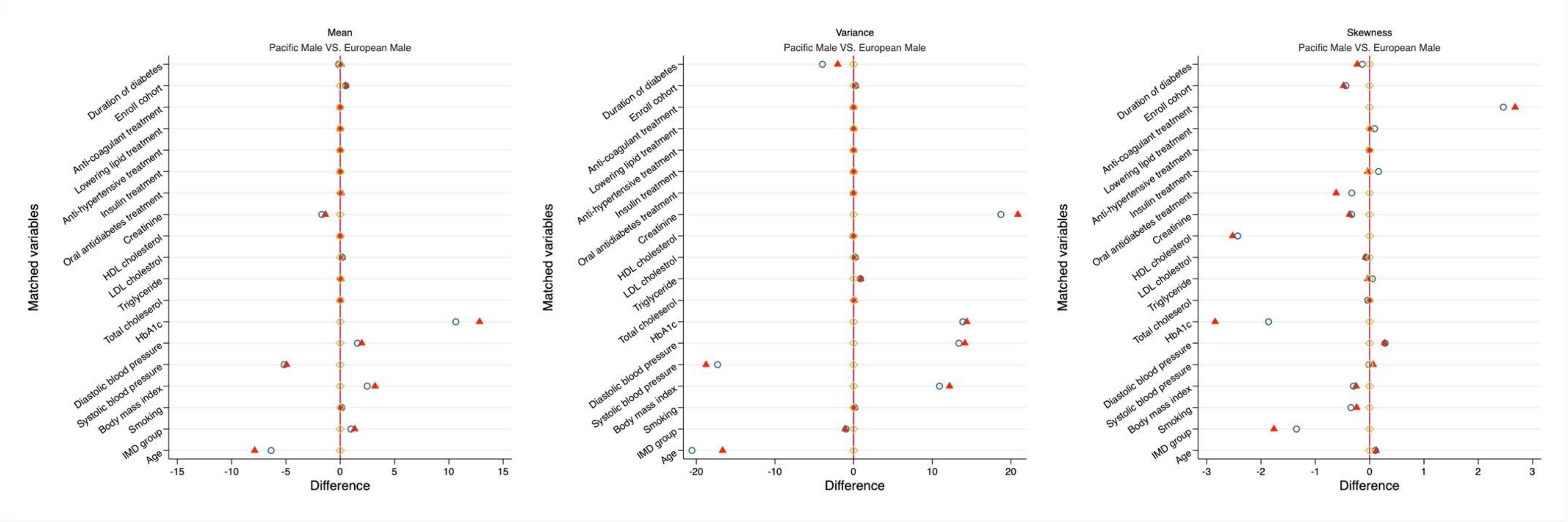

© 2022 Yu D et al. *JAMA Network Open*.

eFigure 10. Adjusted hazard ratios for association between Māori (reference to NZ European) and risks of 14 cancer

Model (i) weighted for age and sex; model (ii) weighted for all adjusted variables in model (i) plus IMD group; model (iii) weighted for all adjusted variables in model (ii) plus smoking status; model (iv) weighted for all adjusted variables in model (iii) plus body measurements (body mass index, systolic and diastolic blood pressure); model (v) weighted for all adjusted variables in model (iv) plus baseline HbA1c; model (vi) weighted for all adjusted variables in model (v) plus baseline lipid profile (total cholesterol, low-density lipoprotein cholesterol, high-density lipoprotein cholesterol, and triglyceride); model (vii) weighted for all adjusted variables in model (vi) plus baseline creatinine; model (viii) weighted for all adjusted variables in model (vii) plus antidiabetes treatments; model (ix) weighted for all adjusted variables in model (viii) plus antihypertensive, lowering lipid and anticoagulant treatment; model (x) weighted for all adjusted variables in model (ix) plus entry cohorts; model (xi) weighted for all adjusted variables in model (x) plus duration of having diabetes at entry time. The log-scale for Y-axis (Hazard ratio) was applied.

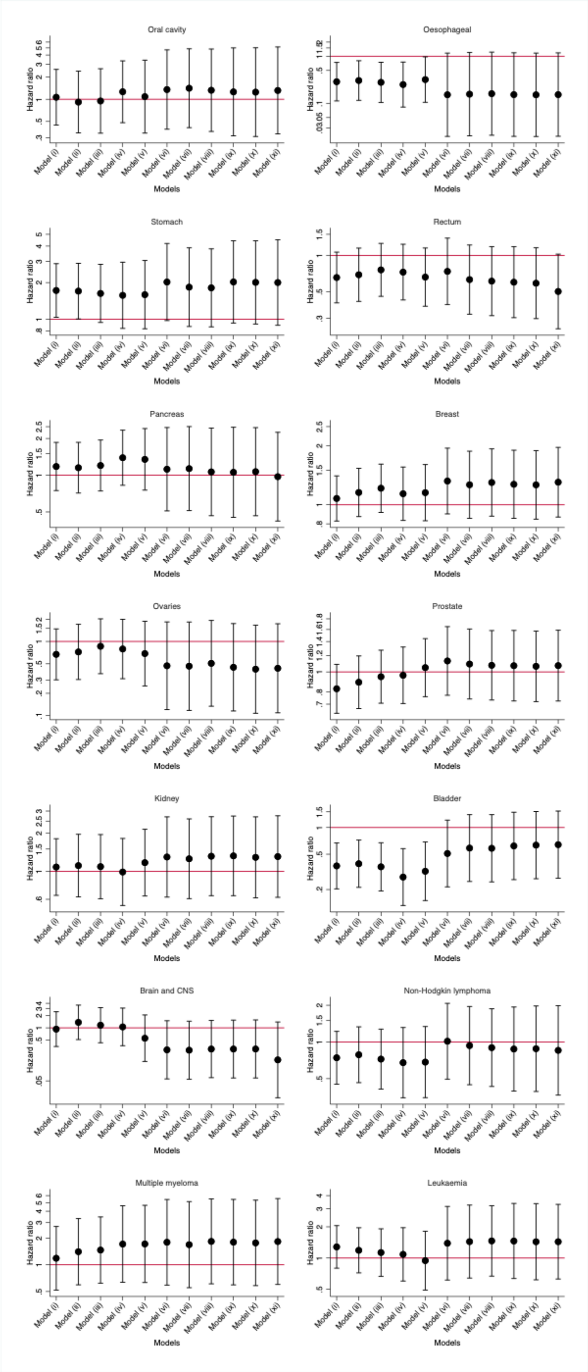

© 2022 Yu D et al. *JAMA Network Open*.

eFigure 11. Adjusted hazard ratios for association between Pasifika (reference to NZ European) and risks of 15 cancer

Model (i) weighted for age and sex; model (ii) weighted for all adjusted variables in model (i) plus IMD group; model (iii) weighted for all adjusted variables in model (ii) plus smoking status; model (iv) weighted for all adjusted variables in model (iii) plus body measurements (body mass index, systolic and diastolic blood pressure); model (v) weighted for all adjusted variables in model (iv) plus baseline HbA1c; model (vi) weighted for all adjusted variables in model (v) plus baseline lipid profile (total cholesterol, low-density lipoprotein cholesterol, high-density lipoprotein cholesterol, and triglyceride); model (vii) weighted for all adjusted variables in model (vi) plus baseline creatinine; model (viii) weighted for all adjusted variables in model (vii) plus antidiabetes treatments; model (ix) weighted for all adjusted variables in model (viii) plus antihypertensive, lowering lipid and anticoagulant treatment; model (x) weighted for all adjusted variables in model (ix) plus entry cohorts; model (xi) weighted for all adjusted variables in model (x) plus duration of having diabetes at entry time. The log-scale for Y-axis (Hazard ratio) was applied.

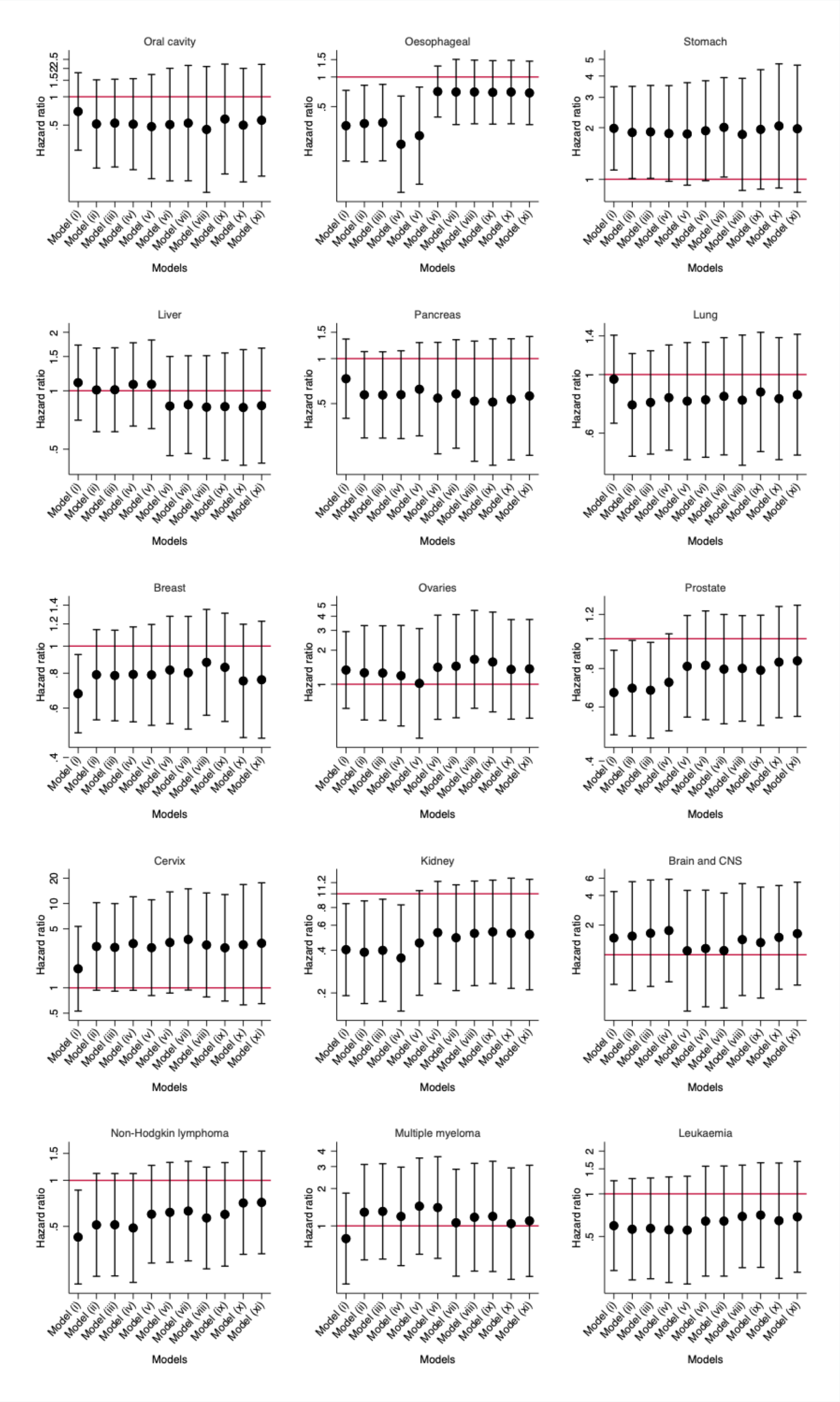

© 2022 Yu D et al. *JAMA Network Open*.

eTable 1. Characteristics in the comparison female cohorts

	Comparison 1: Māori Female vs. European Female								
	**Unmatched cohorts**			**Cohorts after coarsened exact matching**			**Cohorts after entropy matching**		
	NZ European Female	Māori Female	P-value	NZ European Female	Māori Female	P-value	NZ European Female	Māori Female	P-value
	N=6,940	N=3,341		N=3766	N=2488		N=3766	N=2488	
Age, years	63.1 (13.6)	51.0 (12.7)	<0.001	61.1 (13.4)	51.6 (12.6)	<0.001	50.7 (11.9)	50.7 (11.9)	1.000
Enrol cohort, n (%)									
1994-1998	2218 (32.0)	872 (26.1)	<0.001	1232 (32.7)	634 (25.5)	<0.001	15.0 (0.01)	15.1 (0.01)	0.9536
1999-2003	1931 (27.8)	838 (25.1)		1067 (28.3)	652 (26.2)		24.0 (0.02)	23.5 (0.01)	
2004-2008	1357 (19.6)	608 (18.2)		683 (18.1)	437 (17.6)		21.9 (0.02)	22.6 (0.01)	
2009-2013	1044 (15.0)	773 (23.1)		608 (16.1)	599 (24.1)		31.5 (0.02)	31.1 (0.01)	
2014-2018	390 (5.6)	250 (7.5)		176 (4.7)	166 (6.7)		7.6 (0.01)	7.7 (0.01)	
Duration of having diabetes, years	4.0 (1.2)	4.4 (1.4)	<0.001	4.1 (1.2)	4.3 (1.4)	0.036	3.8 (0.8)	3.8 (0.8)	1.000
IMD group (NZDep13 scale)									
IMD-1 (1 or 2)	1233 (17.8)	103 (3.1)	<0.001	529 (14.1)	67 (2.7)	<0.001	2.8 (0.01)	2.8 (0.01)	0.9961
IMD-2 (3 or 4)	1137 (16.4)	218 (6.5)		481 (12.8)	163 (6.5)		6.3 (0.01)	6.3 (0.01)	
IMD-3 (5 or 6)	1085 (15.6)	325 (9.7)		453 (12.0)	231 (9.3)		8.4 (0.01)	8.4 (0.01)	
IMD-4 (7 or 8)	1906 (27.5)	801 (24.0)		1239 (32.9)	618 (24.9)		26.0 (0.02)	26.0 (0.02)	
IMD-5 (9 or 10)	1578 (22.7)	1895 (56.7)		1065 (28.3)	1409 (56.6)		56.6 (0.02)	56.6 (0.02)	
Smoking status, n (%)									
Never smoking	5182 (74.7)	1544 (46.2)	<0.001	2780 (73.8)	1237 (49.7)	<0.001	41.0 (0.02)	41.0 (0.02)	1.00
Ex-smoker	1058 (15.2)	716 (21.4)		571 (15.2)	517 (20.8)		25.5 (0.02)	25.5 (0.02)	
Current Smoker	700 (10.1)	1081 (32.4)		415 (11.0)	734 (29.5)		33.5 (0.02)	33.5 (0.02)	
Body mass index, kg/m^2	31.7 (7.1)	36.2 (7.9)	<0.001	32.8 (7.2)	36.5 (7.7)	<0.001	36.9 (7.7)	36.9 (7.7)	1.000
Systolic blood pressure, mmHg	139 (18)	135 (19)	<0.001	140 (18)	136 (19)	<0.001	135 (18)	135 (18)	1.000
Diastolic blood pressure, mmHg	79 (10)	82 (11)	<0.001	80 (10)	83 (11)	<0.001	83 (11)	83 (11)	1.000
HbA1c, mmol/mol	54.2 (17.0)	65 (22.1)	<0.001	55.3 (16.0)	64.7 (21.3)	<0.001	63.4 (20.2)	63.4 (20.2)	1.000
Total cholesterol, mmol/L	5.4 (1.1)	5.2 (1.1)	<0.001	5.4 (1.2)	5.3 (1.2)	0.980	5.2 (1.1)	5.2 (1.1)	1.000

© 2022 Yu D et al. *JAMA Network Open*.

Triglyceride, mmol/L	2.2 (1.3)	2.5 (1.7)	<0.001	2.3 (1.4)	2.5 (1.5)	<0.001	2.4 (1.4)	2.4 (1.4)	1.000
Low-density lipoprotein cholesterol, mmol/L	2.6 (0.9)	2.6 (1.0)	<0.001	2.6 (0.9)	2.7 (1.0)	<0.001	2.7 (1.0)	2.7 (1.0)	1.000
High-density lipoprotein cholesterol, mmol/L	1.3 (0.4)	1.1 (0.3)	<0.001	1.3 (0.4)	1.2 (0.3)	<0.001	1.2 (0.3)	1.2 (0.3)	1.000
Creatinine, mmol/L	78.2 (17.3)	73.4 (17.5)	<0.001	76.3 (17.0)	72.3 (16.7)	<0.001	70.4 (15.5)	70.4 (15.5)	1.000
Antidiabetes treatment, n (%)									
Oral antidiabetes drug and insulin	1052 (15.2)	702 (21.0)	<0.001	760 (20.2)	590 (23.7)	<0.001	25.5 (0.02)	25.9 (0.01)	0.3962
Oral antidiabetes drug only	3763 (54.2)	1932 (57.8)		2239 (59.5)	1510 (60.7)		63.9 (0.02)	63.5 (0.01)	
Insulin only	269 (3.9)	111 (3.3)		147 (3.9)	79 (3.2)		2.4 (0.01)	2.1 (0.01)	
Antihypertensive treatment, n (%)	4759 (68.6)	2337 (70.0)	<0.001	2889 (76.7)	1912 (76.9)	0.7653	83.8 (0.02)	83.8 (0.02)	1.0000
Statin treatment, n (%)	3705 (53.4)	1931 (57.8)	<0.001	2363 (62.8)	1609 (64.7)	0.085	80.8 (0.02)	80.8 (0.02)	1.0000
Antiplatelet or anticoagulant treatment, n (%)	191 (2.8)	79 (2.4)	<0.001	114 (3.0)	63 (2.5)	0.036	3.4 (0.01)	3.4 (0.01)	0.9999
	**Comparison 2: Pacific Female vs. European Female**								
	NZ European Female	Pasifika Female		NZ European Female	Pasifika Female		NZ European Female	Pasifika Female	
	N=6,940	N=5,984		N=4,455	N=4,612		N=4,455	N=4,612	
Age, years	63.1 (13.6)	52.9 (13.3)	<0.001	62.0 (13.5)	53.3 (13.2)	<0.001	52.0 (12.7)	52.0 (12.7)	1.000
Enrol cohort, n (%)									
1994-1998	2218 (32.0)	1367 (22.8)	<0.001	1500 (33.7)	1029 (22.3)	<0.001	12.9 (0.01)	12.2 (0.01)	0.1062
1999-2003	1931 (27.8)	1780 (29.8)		1173 (26.3)	1405 (30.5)		18.9 (0.02)	18.4 (0.01)	
2004-2008	1357 (19.6)	715 (12.0)		864 (19.4)	539 (11.7)		19.8 (0.02)	19.1(0.01)	
2009-2013	1044 (15.0)	1368 (22.9)		707 (15.9)	1108 (24.0)		32.5 (0.02)	33.9 (0.01)	
2014-2018	390 (5.6)	754 (12.6)		211 (4.7)	531 (11.5)		15.9 (0.02)	15.3 (0.01)	
Duration of having diabetes, years	4.0 (1.2)	4.5 (5.3)	<0.001	4.1 (1.3)	4.4 (1.3)	<0.001	4.1 (0.9)	4.1(0.9)	1.000
IMD group (NZDep13 scale)									
IMD-1 (1 or 2)	1233 (17.8)	158 (2.6)	<0.001	641 (14.4)	119 (2.6)	<0.001	3.1 (0.004)	2.8 (0.003)	0.4632
IMD-2 (3 or 4)	1137 (16.4)	283 (4.7)		590 (13.3)	214 (4.7)		3.8 (0.003)	4.0 (0.004)	
IMD-3 (5 or 6)	1085 (15.6)	328 (5.5)		543 (12.2)	240 (5.2)		7.1 (0.01)	5.2 (0.004)	
IMD-4 (7 or 8)	1906 (27.5)	1269 (21.2)		1459 (32.8)	992 (21.5)		20.6 (0.01)	21.9 (0.01)	
IMD-5 (9 or 10)	1578 (22.7)	3946 (66.0)		1222 (27.4)	3048 (66.1)		65.4 (0.02)	65.1 (0.01)	
Smoking status, n (%)									
Never smoking	5182 (74.7)	4777 (79.8)	<0.001	3623 (81.3)	3780 (82.0)	<0.001	76.6 (0.02)	76.6 (0.02)	0.9999
Ex-smoker	1058 (15.2)	610 (10.2)		478 (10.7)	432 (9.4)		12.8 (0.01)	12.8 (0.01)	

© 2022 Yu D et al. *JAMA Network Open*.

Current Smoker	700 (10.1)	597 (10.0)		354 (7.9)	400 (8.7)		10.6 (0.01)	10.6 (0.01)	
Body mass index, kg/m^2	31.7 (7.1)	35.6 (7.5)	<0.001	32.5 (7.1)	35.8 (7.5)		36.0 (7.8)	36.0 (7.8)	1.000
Systolic blood pressure, mmHg	139 (19)	133 (18)	<0.001	140 (18)	135 (18)	<0.001	133 (17)	133 (17)	1.000
Diastolic blood pressure, mmHg	83 (11)	80 (12)	<0.001	82 (10)	80 (10)	<0.001	82 (10)	82 (10)	1.000
HbA1c, mmol/mol	53.6 (16.1)	67.1 (21.7)	<0.001	55.1 (15.7)	67.3 (21.3)	<0.001	65.5 (20.3)	65.5 (20.3)	1.000
Total cholesterol, mmol/L	5.4 (1.2)	5.1 (1.2)	<0.001	5.4 (1.2)	5.1 (1.2)	0.003	5.0 (1.1)	5.0 (1.1)	1.000
Triglyceride, mmol/L	2.2 (1.3)	2.0 (1.5)	<0.001	2.3 (1.4)	2.1 (1.5)	0.003	1.9 (1.2)	1.9 (1.2)	1.000
Low-density lipoprotein cholesterol, mmol/L	2.6 (0.9)	2.6 (0.9)	<0.001	2.6 (0.9)	2.6 (0.9)	0.002	2.6 (0.9)	2.6 (0.9)	1.000
High-density lipoprotein cholesterol, mmol/L	1.3 (0.4)	1.2 (0.4)	<0.001	1.3 (0.4)	1.2 (0.4)	0.003	1.2 (0.4)	1.2 (0.4)	1.000
Creatinine, mmol/L	78.2 (17.3)	74.1 (18.5)	<0.001	76.8 (17.2)	73.0 (18.4)	0.004	70.9 (17.1)	70.9 (17.1)	1.000
Antidiabetes treatment, n (%)									
Oral antidiabetes drug and insulin	1052 (15.2)	1125 (18.8)	<0.001	848 (19.0)	958 (20.8)	<0.001	24.9 (0.02)	25.2 (0.01)	0.3027
Oral antidiabetes drug only	3763 (54.2)	3691 (61.7)		2674 (60.0)	2900 (62.9)		68.6 (0.02)	68.3 (0.01)	
Insulin only	269 (3.9)	158 (2.6)		175 (3.9)	96 (2.1)		2.1 (0.01)	2.5 (0.01)	
Antihypertensive treatment, n (%)	4759 (68.6)	3845 (64.3)	<0.001	3299 (74.1)	3231 (70.1)	0.001	81.9 (0.02)	81.9 (0.02)	1.000
Statin treatment, n (%)	3705 (53.4)	2924 (48.9)	<0.001	2652 (59.5)	2487 (53.9)	0.003	75.4 (0.02)	75.4 (0.02)	1.000
Antiplatelet or anticoagulant treatment, n (%)	191 (2.8)	77 (1.3)	<0.001	139 (3.1)	62 (1.3)	0.001	1.9 (0.01)	1.9 (0.01)	1.000

© 2022 Yu D et al. *JAMA Network Open*.

eTable 2. Characteristics in the comparison male cohorts

	Comparison 1: Māori Male vs. European Male								
	**Unmatched cohorts**			**Cohorts after coarsened exact matching**			**Cohorts after entropy matching**		
	NZ European male	Māori male	P-value	NZ European male	Māori male	P-value	NZ European male	Māori male	P-value
	N=8522	N=3311		N=4595	N=2542		N=4595	N=2542	
Age, years	60.4 (12.7)	51.0 (12.0)	<0.001	58.9 (12.4)	51.2 (12.0)	<0.001	50.6 (11.6)	50.6 (11.6)	1.000
Enrol cohort, n (%)									
1994-1998	2493 (29.3)	827 (25.0)	<0.001	1416 (30.8)	624 (24.6)	<0.001	14.7 (0.01)	14.1 (0.01)	0.1031
1999-2003	2306 (27.1)	756 (22.8)		1148 (25.0)	584 (23.0)		19.3 (0.01)	19.6 (0.01)	
2004-2008	1697 (19.9)	621 (18.8)		900 (19.6)	479 (18.8)		27.5 (0.01)	26.2 (0.01)	
2009-2013	1382 (16.2)	782 (23.6)		818 (17.8)	626 (24.6)		28.3 (0.02)	30.5 (0.01)	
2014-2018	644 (7.6)	325 (9.8)		313 (6.8)	229 (9.0)		10.2 (0.01)	9.6 (0.01)	
Duration of having diabetes, years	3.9 (1.0)	4.1 (1.2)	<0.001	4.1 (1.1)	4.1 (1.1)	0.0783	3.6 (0.7)	3.6 (0.7)	1.000
IMD group (NZDep13 scale)									
IMD-1 (1 or 2)	1731 (20.3)	152 (4.6)	<0.001	779 (17.0)	128 (5.0)	<0.001	5.0 (0.01)	5.2 (0.01)	0.2312
IMD-2 (3 or 4)	1514 (17.8)	274 (8.3)		708 (15.4)	217 (8.5)		10.0 (0.01)	9.3 (0.01)	
IMD-3 (5 or 6)	1334 (15.7)	354 (10.7)		606 (13.2)	261 (10.3)		9.5 (0.01)	10.4 (0.01)	
IMD-4 (7 or 8)	2146 (25.2)	768 (23.2)		1348 (29.3)	596 (23.4)		23.7 (0.01)	23.1 (0.01)	
IMD-5 (9 or 10)	1796 (21.1)	1762 (53.2)		1155 (25.1)	1340 (52.7)		51.9 (0.02)	52.0 (0.01)	
Smoking status, n (%)									
Never smoking	5358 (62.9)	1550 (46.8)	<0.001	2794 (60.8)	1221 (48.0)	<0.001	42.2 (0.02)	42.2 (0.02)	0.9999
Ex-smoker	2166 (25.4)	839 (25.3)		1228 (26.7)	653 (25.7)		30.3 (0.02)	30.3 (0.02)	
Current Smoker	998 (11.7)	922 (27.9)		573 (12.5)	668 (26.3)		27.5 (0.02)	27.5 (0.02)	
Body mass index, kg/m^2	30.7 (5.8)	35.4 (7.0)	<0.001	31.6 (5.9)	35.5 (7.0)	<0.001	35.8 (7.0)	35.8 (7.0)	1.000
Systolic blood pressure, mmHg	136 (17)	135 (19)	<0.001	138 (16)	136 (18)	<0.001	134 (18)	134 (18)	1.000
Diastolic blood pressure, mmHg	80 (10)	85 (12)	<0.001	81 (10)	85 (12)	<0.001	84 (12)	84 (12)	1.000
HbA1c, mmol/mol	54.7 (18.1)	65.5 (21.6)	<0.001	56.5 (17.4)	64.4 (20.2)	<0.001	64.4 (20.2)	64.4 (20.2)	1.000
Total cholesterol, mmol/L	4.9 (1.1)	5.1 (1.2)	<0.001	5.0 (1.2)	5.2 (1.2)	0.980	5.0 (1.2)	5.0 (1.2)	1.000
Triglyceride, mmol/L	2.2 (1.6)	2.8 (1.1)	<0.001	2.4 (1.6)	2.8 (2.1)	<0.001	2.6 (1.8)	2.6 (1.8)	1.000

© 2022 Yu D et al. *JAMA Network Open*.

Low-density lipoprotein cholesterol, mmol/L	2.4 (0.9)	2.5 (1.0)	<0.001	2.4 (0.9)	2.6 (1.0)	<0.001	2.6 (1.0)	2.6 (1.0)	1.000
High-density lipoprotein cholesterol, mmol/L	1.1 (0.4)	1.1 (0.3)	<0.001	1.1 (0.4)	1.1 (0.3)	<0.001	1.1 (0.3)	1.1 (0.3)	1.000
Creatinine, mmol/L	91.6 (17.1)	87.4 (17.6)	<0.001	91.9 (16.9)	87.8 (17.5)	<0.001	86.8 (16.7)	86.8 (16.7)	1.000
Antidiabetes treatment, n (%)									
Oral antidiabetes drug and insulin	1346 (15.8)	646 (19.5)	<0.001	982 (21.4)	579 (22.8)	<0.001	25.9 (0.01)	26.6 (0.01)	0.2318
Oral antidiabetes drug only	4874 (57.2)	1982 (59.9)		2867 (62.4)	1592 (62.6)		65.9 (0.01)	65.2 (0.01)	
Insulin only	301 (3.5)	94 (2.8)		162 (3.5)	58 (2.3)		2.2 (0.01)	1.5 (0.01)	
Antihypertensive treatment, n (%)	5916 (69.4)	2390 (72.2)	<0.001	3583 (78.0)	2015 (79.3)	0.0731	89.8 (0.01)	89.8 (0.01)	1.000
Statin treatment, n (%)	4850 (56.9)	1959 (59.2)	<0.001	3061 (66.6)	1711 (67.3)	0.085	83.1 (0.01)	83.1 (0.01)	1.000
Antiplatelet or anticoagulant treatment, n (%)	324 (3.8)	107 (3.2)	<0.001	208 (4.5)	95 (3.7)	0.036	5.0 (0.01)	5.0 (0.01)	1.000
	**Comparison 1: Pacific Male vs. European Male**								
	NZ European male	Pasifika male	P-value	NZ European male	Pasifika male	P-value	NZ European male	Pasifika male	P-value
	N=8522	5405		N=4885	N=4216		N=4885	N=4216	
Age, years	60.4 (12.7)	52.5 (12.0)	<0.001	59.2 (12.6)	52.9 (11.8)	<0.001	51.9 (11.1)	51.9 (11.1)	1.000
Enrol cohort, n (%)									
1994-1998	2493 (29.3)	1015 (18.8)	<0.001	1465 (30.0)	765 (18.2)	<0.001	10.1 (0.01)	9.3 (0.01)	0.1748
1999-2003	2306 (27.1)	1304 (24.1)		1164 (23.8)	999 (23.7)		13.1 (0.01)	13.3 (0.01)	
2004-2008	1697 (19.9)	766 (14.2)		1055 (21.6)	590 (14.0)		22.1 (0.02)	20.4 (0.02)	
2009-2013	1382 (16.2)	1514 (28.0)		868 (17.8)	1246 (29.6)		36.3 (0.02)	39.4 (0.02)	
2014-2018	644 (7.6)	806 (14.9)		333 (6.8)	616 (14.6)		18.4 (0.02)	17.6 (0.02)	
Duration of having diabetes, years	3.9 (1.0)	3.9 (1.7)	<0.001	4.1 (1.1)	4.0 (1.7)		4.0 (1.6)	4.0 (1.6)	1.000
IMD group (NZDep13 scale)									
IMD-1 (1 or 2)	1731 (20.3)	157 (2.9)	<0.001	734 (15.0)	121 (2.9)	<0.001	3.0 (0.003)	2.9 (0.003)	0.3900
IMD-2 (3 or 4)	1514 (17.8)	276 (5.1)		673 (13.8)	219 (5.2)		5.1 (0.01)	5.6 (0.01)	
IMD-3 (5 or 6)	1334 (15.7)	285 (5.3)		559 (11.5)	233 (5.5)		5.5 (0.01)	4.8 (0.01)	
IMD-4 (7 or 8)	2146 (25.2)	1129 (20.9)		1595 (32.7)	879 (20.9)		20.6 (0.01)	21.1 (0.01)	
IMD-5 (9 or 10)	1796 (21.1)	3558 (65.8)		1323 (27.1)	2764 (65.6)		65.9 (0.02)	65.7 (0.02)	
Smoking status, n (%)									
Never smoking	5358 (62.9)	3228 (59.7)	<0.001	3228 (66.1)	2538 (60.2)	<0.001	54.4 (0.02)	54.4 (0.02)	0.9999
Ex-smoker	2166 (25.4)	1127 (20.9)		1132 (23.2)	891 (21.1)		25.6 (0.02)	25.6 (0.02)	
Current Smoker	998 (11.7)	1050 (19.4)		525 (10.8)	787 (18.7)		20.0 (0.01)	20.0 (0.01)	
Body mass index, kg/m^2	30.7 (5.8)	33.9 (6.8)	<0.001	31.4 (5.9)	33.9 (6.8)		34.1 (6.9)	34.1 (6.9)	1.000

© 2022 Yu D et al. *JAMA Network Open*.

Systolic blood pressure, mmHg	136 (17)	136 (17)	<0.001	137 (17)	132 (16)	<0.001	131 (16)	131 (16)	1.000
Diastolic blood pressure, mmHg	80 (10)	82 (11)	<0.001	81 (10)	82 (11)	<0.001	82 (11)	82 (11)	1.000
HbA1c, mmol/mol	54.7 (18.1)		<0.001	57.4 (18.1)	68.0 (21.6)	<0.001	66.5 (20.8)	66.5 (20.8)	1.000
Total cholesterol, mmol/L	4.9 (1.1)	5.0 (1.2)	<0.001	5.0 (1.2)	5.0 (1.2)	0.003	4.8 (1.2)	4.8 (1.2)	1.000
Triglyceride, mmol/L	2.2 (1.6)	2.3 (1.8)	<0.001	2.3 (1.5)	2.3 (1.8)	0.1032	2.1 (1.4)	2.1 (1.4)	1.000
Low-density lipoprotein cholesterol, mmol/L	2.4 (0.9)	2.6 (1.0)	<0.001	2.4 (0.9)	2.6 (1.0)	0.0216	2.6 (1.0)	2.6 (1.0)	1.000
High-density lipoprotein cholesterol, mmol/L	1.1 (0.4)	1.1 (0.3)	<0.001	1.1 (0.4)	1.1 (0.3)	0.0328	1.1 (0.3)	1.1 (0.3)	1.000
Creatinine, mmol/L	91.6 (17.1)	90.2 (18.5)	<0.001	92.4 (16.6)	90.7 (18.3)	0.004	89.2 (17.7)	89.2 (17.7)	1.000
Antidiabetes treatment, n (%)									
Oral antidiabetes drug and insulin	1346 (15.8)	944 (17.5)	<0.001	993 (20.3)	827 (19.6)	<0.001	22.0 (0.01)	22.8 (0.01)	0.5321
Oral antidiabetes drug only	4874 (57.2)	3479 (64.4)		3113 (63.7)	2837 (67.3)		71.5 (0.01)	71.0 (0.01)	
Insulin only	301 (3.5)	124 (2.3)		176 (3.6)	80 (1.9)		1.7 (0.01)	1.2 (0.01)	
Antihypertensive treatment, n (%)	5916 (69.4)	3751 (69.4)	<0.001	3689 (75.5)	3189 (75.6)	0.0632	86.2 (0.01)	86.2 (0.01)	1.000
Statin treatment, n (%)	4850 (56.9)	3058 (56.6)	<0.001	3164 (64.8)	2644 (62.7)	0.003	81.4 (0.01)	81.4 (0.01)	1.000
Antiplatelet or anticoagulant treatment, n (%)	324 (3.8)	91 (1.7)	<0.001	222 (4.5)	85 (2.0)	0.001	2.6 (0.01)	2.6 (0.01)	1.000

© 2022 Yu D et al. *JAMA Network Open*.

eTable 3. Adjusted hazard ratios for association between Māori (reference to NZ European) and risks of 21 cancer

Model (i) weighted for age and sex; model (ii) weighted for all adjusted variables in model (i) plus IMD group; model (iii) weighted for all adjusted variables in model (ii) plus smoking status; model (iv) weighted for all adjusted variables in model (iii) plus body measurements (body mass index, systolic and diastolic blood pressure); model (v) weighted for all adjusted variables in model (iv) plus baseline HbA1c; model (vi) weighted for all adjusted variables in model (v) plus baseline lipid profile (total cholesterol, low-density lipoprotein cholesterol, high-density lipoprotein cholesterol, and triglyceride); model (vii) weighted for all adjusted variables in model (vi) plus baseline creatinine; model (viii) weighted for all adjusted variables in model (vii) plus antidiabetes treatments; model (ix) weighted for all adjusted variables in model (viii) plus antihypertensive, lowering lipid and anticoagulant treatment; model (x) weighted for all adjusted variables in model (ix) plus entry cohorts; model (xi) weighted for all adjusted variables in model (x) plus duration of having diabetes at entry time.

Model	Cancer Type	Hazard Ratio (95% Confidence Interval)
I	Bladder	0.37 (0.20-0.67)
II	Bladder	0.39 (0.21-0.72)
III	Bladder	0.36 (0.19-0.67)
IV	Bladder	0.28 (0.13-0.58)
V	Bladder	0.32 (0.15-0.69)
VI	Bladder	0.51 (0.21-1.21)
VII	Bladder	0.59 (0.25-1.39)
VIII	Bladder	0.58 (0.24-1.39)
IX	Bladder	0.62 (0.26-1.49)
X	Bladder	0.63 (0.26-1.51)
XI	Bladder	0.64 (0.27-1.53)
I	Brain and CNS	0.93 (0.35-2.48)
II	Brain and CNS	1.36 (0.52-3.61)
III	Brain and CNS	1.16 (0.43-3.14)
IV	Brain and CNS	1.06 (0.37-3.05)
V	Brain and CNS	0.56 (0.15-2.07)
VI	Brain and CNS	0.29 (0.06-1.49)
VII	Brain and CNS	0.28 (0.06-1.46)
VIII	Brain and CNS	0.30 (0.06-1.53)
IX	Brain and CNS	0.30 (0.06-1.55)
X	Brain and CNS	0.30 (0.06-1.56)
XI	Brain and CNS	0.16 (0.02-1.39)
I	Breast	1.08 (0.83-1.40)
II	Breast	1.16 (0.87-1.53)
III	Breast	1.21 (0.91-1.61)
IV	Breast	1.14 (0.83-1.56)
V	Breast	1.15 (0.83-1.60)
VI	Breast	1.32 (0.90-1.94)
VII	Breast	1.26 (0.85-1.87)
VIII	Breast	1.30 (0.88-1.93)
IX	Breast	1.27 (0.85-1.90)
X	Breast	1.26 (0.85-1.89)

XI	Breast	1.30 (0.87-1.96)
I	Cervix	1.25 (0.44-3.58)
II	Cervix	1.67 (0.57-4.87)
III	Cervix	1.65 (0.52-5.31)
IV	Cervix	1.45 (0.41-5.11)
V	Cervix	1.72 (0.48-6.16)
VI	Cervix	2.18 (0.50-9.53)
VII	Cervix	5.08 (1.22-21.23)
VIII	Cervix	4.79 (1.13-20.40)
IX	Cervix	4.54 (1.03-20.05)
X	Cervix	4.54 (1.03-19.95)
XI	Cervix	4.81 (1.08-21.42)
I	Colon	0.49 (0.35-0.69)
II	Colon	0.50 (0.35-0.71)
III	Colon	0.49 (0.34-0.70)
IV	Colon	0.46 (0.31-0.68)
V	Colon	0.48 (0.32-0.72)
VI	Colon	0.57 (0.36-0.90)
VII	Colon	0.55 (0.34-0.87)
VIII	Colon	0.55 (0.35-0.87)
IX	Colon	0.54 (0.34-0.87)
X	Colon	0.54 (0.34-0.87)
XI	Colon	0.56 (0.35-0.90)
I	Gallbladder	4.88 (1.59-14.96)
II	Gallbladder	6.38 (2.17-18.78)
III	Gallbladder	6.95 (2.32-20.80)
IV	Gallbladder	6.38 (2.03-20.06)
V	Gallbladder	5.44 (1.70-17.44)
VI	Gallbladder	7.14 (1.57-32.57)
VII	Gallbladder	7.02 (1.52-32.45)
VIII	Gallbladder	6.73 (1.48-30.48)
IX	Gallbladder	7.52 (1.52-37.29)
X	Gallbladder	7.93 (1.59-39.47)
XI	Gallbladder	7.94 (1.57-40.24)
I	Kidney	1.08 (0.64-1.81)
II	Kidney	1.11 (0.63-1.96)
III	Kidney	1.09 (0.61-1.96)
IV	Kidney	0.99 (0.54-1.82)
V	Kidney	1.17 (0.64-2.15)
VI	Kidney	1.30 (0.62-2.70)
VII	Kidney	1.26 (0.61-2.60)
VIII	Kidney	1.32 (0.64-2.71)

© 2022 Yu D et al. *JAMA Network Open*.

IX	Kidney	1.33 (0.64-2.74)
X	Kidney	1.29 (0.62-2.70)
XI	Kidney	1.31 (0.62-2.75)
I	Leukaemia	1.28 (0.80-2.05)
II	Leukaemia	1.19 (0.72-1.96)
III	Leukaemia	1.13 (0.67-1.91)
IV	Leukaemia	1.09 (0.60-1.96)
V	Leukaemia	0.95 (0.49-1.81)
VI	Leukaemia	1.39 (0.61-3.14)
VII	Leukaemia	1.44 (0.64-3.24)
VIII	Leukaemia	1.46 (0.67-3.20)
IX	Leukaemia	1.46 (0.63-3.36)
X	Leukaemia	1.44 (0.62-3.35)
XI	Leukaemia	1.44 (0.63-3.29)
I	Liver	1.65 (1.12-2.43)
II	Liver	1.64 (1.05-2.55)
III	Liver	1.56 (0.98-2.49)
IV	Liver	1.80 (1.17-2.75)
V	Liver	1.98 (1.30-3.02)
VI	Liver	1.83 (1.11-3.01)
VII	Liver	1.76 (1.06-2.93)
VIII	Liver	1.78 (1.08-2.94)
IX	Liver	1.88 (1.13-3.14)
X	Liver	1.89 (1.13-3.15)
XI	Liver	1.81 (1.08-3.03)
I	Lung	2.97 (2.33-3.79)
II	Lung	2.69 (2.04-3.55)
III	Lung	2.13 (1.60-2.83)
IV	Lung	2.15 (1.56-2.96)
V	Lung	2.21 (1.53-3.21)
VI	Lung	1.81 (1.14-2.85)
VII	Lung	1.80 (1.14-2.85)
VIII	Lung	1.75 (1.08-2.84)
IX	Lung	1.99 (1.30-3.04)
X	Lung	2.01 (1.32-3.06)
XI	Lung	1.97 (1.30-2.99)
I	Malignant melanoma	0.08 (0.04-0.18)
II	Malignant melanoma	0.08 (0.04-0.18)
III	Malignant melanoma	0.09 (0.04-0.20)
IV	Malignant melanoma	0.11 (0.05-0.23)
V	Malignant melanoma	0.12 (0.05-0.26)
VI	Malignant melanoma	0.12 (0.05-0.28)

© 2022 Yu D et al. *JAMA Network Open*.

VII	Malignant melanoma	0.10 (0.04-0.26)
VIII	Malignant melanoma	0.10 (0.04-0.26)
IX	Malignant melanoma	0.10 (0.04-0.26)
X	Malignant melanoma	0.10 (0.04-0.26)
XI	Malignant melanoma	0.11 (0.04-0.27)
I	Multiple myeloma	1.18 (0.52-2.69)
II	Multiple myeloma	1.40 (0.59-3.31)
III	Multiple myeloma	1.46 (0.62-3.45)
IV	Multiple myeloma	1.71 (0.63-4.60)
V	Multiple myeloma	1.71 (0.63-4.66)
VI	Multiple myeloma	1.79 (0.59-5.42)
VII	Multiple myeloma	1.68 (0.55-5.13)
VIII	Multiple myeloma	1.83 (0.61-5.49)
IX	Multiple myeloma	1.80 (0.59-5.43)
X	Multiple myeloma	1.76 (0.58-5.33)
XI	Multiple myeloma	1.83 (0.60-5.56)
I	Non-Hodgkin lymphoma	0.74 (0.45-1.22)
II	Non-Hodgkin lymphoma	0.79 (0.46-1.33)
III	Non-Hodgkin lymphoma	0.72 (0.41-1.27)
IV	Non-Hodgkin lymphoma	0.68 (0.35-1.31)
V	Non-Hodgkin lymphoma	0.68 (0.35-1.34)
VI	Non-Hodgkin lymphoma	1.01 (0.50-2.07)
VII	Non-Hodgkin lymphoma	0.93 (0.45-1.95)
VIII	Non-Hodgkin lymphoma	0.90 (0.43-1.87)
IX	Non-Hodgkin lymphoma	0.88 (0.40-1.93)
X	Non-Hodgkin lymphoma	0.88 (0.39-1.97)
XI	Non-Hodgkin lymphoma	0.85 (0.37-1.99)
I	Oesophageal	0.29 (0.11-0.74)
II	Oesophageal	0.31 (0.12-0.80)
III	Oesophageal	0.28 (0.10-0.74)
IV	Oesophageal	0.25 (0.08-0.75)
V	Oesophageal	0.32 (0.11-0.97)
VI	Oesophageal	0.15 (0.02-1.17)
VII	Oesophageal	0.16 (0.02-1.20)
VIII	Oesophageal	0.16 (0.02-1.22)
IX	Oesophageal	0.15 (0.02-1.18)
X	Oesophageal	0.15 (0.02-1.17)
XI	Oesophageal	0.15 (0.02-1.18)
I	Oral cavity	1.07 (0.45-2.55)
II	Oral cavity	0.92 (0.35-2.41)
III	Oral cavity	0.95 (0.35-2.61)
IV	Oral cavity	1.27 (0.48-3.33)

© 2022 Yu D et al. *JAMA Network Open*.

V	Oral cavity	1.09 (0.35-3.41)
VI	Oral cavity	1.36 (0.39-4.70)
VII	Oral cavity	1.41 (0.41-4.86)
VIII	Oral cavity	1.33 (0.36-4.83)
IX	Oral cavity	1.26 (0.32-5.00)
X	Oral cavity	1.25 (0.31-5.02)
XI	Oral cavity	1.32 (0.34-5.14)
I	Ovaries	0.67 (0.30-1.48)
II	Ovaries	0.72 (0.31-1.70)
III	Ovaries	0.86 (0.37-2.02)
IV	Ovaries	0.79 (0.31-1.99)
V	Ovaries	0.69 (0.25-1.87)
VI	Ovaries	0.47 (0.12-1.83)
VII	Ovaries	0.46 (0.12-1.83)
VIII	Ovaries	0.51 (0.13-1.92)
IX	Ovaries	0.45 (0.12-1.74)
X	Ovaries	0.42 (0.11-1.66)
XI	Ovaries	0.43 (0.11-1.73)
I	Pancreas	1.18 (0.75-1.86)
II	Pancreas	1.15 (0.71-1.86)
III	Pancreas	1.20 (0.74-1.94)
IV	Pancreas	1.39 (0.83-2.34)
V	Pancreas	1.35 (0.75-2.41)
VI	Pancreas	1.12 (0.51-2.46)
VII	Pancreas	1.13 (0.51-2.50)
VIII	Pancreas	1.06 (0.47-2.44)
IX	Pancreas	1.06 (0.45-2.47)
X	Pancreas	1.07 (0.47-2.46)
XI	Pancreas	0.97 (0.42-2.25)
I	Prostate	0.83 (0.63-1.09)
II	Prostate	0.89 (0.67-1.19)
III	Prostate	0.95 (0.71-1.27)
IV	Prostate	0.96 (0.70-1.32)
V	Prostate	1.05 (0.76-1.45)
VI	Prostate	1.13 (0.77-1.65)
VII	Prostate	1.09 (0.74-1.61)
VIII	Prostate	1.08 (0.73-1.58)
IX	Prostate	1.07 (0.72-1.58)
X	Prostate	1.06 (0.72-1.57)
XI	Prostate	1.07 (0.72-1.59)
I	Rectum	0.66 (0.40-1.07)
II	Rectum	0.69 (0.42-1.15)

© 2022 Yu D et al. *JAMA Network Open*.

III	Rectum	0.76 (0.46-1.26)
IV	Rectum	0.73 (0.43-1.24)
V	Rectum	0.66 (0.38-1.15)
VI	Rectum	0.74 (0.39-1.39)
VII	Rectum	0.63 (0.33-1.22)
VIII	Rectum	0.61 (0.32-1.19)
IX	Rectum	0.60 (0.30-1.18)
X	Rectum	0.59 (0.30-1.16)
XI	Rectum	0.50 (0.25-1.03)
I	Stomach	1.72 (1.04-2.87)
II	Stomach	1.70 (1.00-2.89)
III	Stomach	1.63 (0.94-2.83)
IV	Stomach	1.57 (0.84-2.95)
V	Stomach	1.59 (0.83-3.04)
VI	Stomach	2.02 (0.97-4.20)
VII	Stomach	1.84 (0.87-3.87)
VIII	Stomach	1.81 (0.86-3.79)
IX	Stomach	2.03 (0.93-4.42)
X	Stomach	2.01 (0.91-4.43)
XI	Stomach	2.00 (0.89-4.51)
I	Thyroid	7.03 (2.91-16.98)
II	Thyroid	6.83 (2.48-18.79)
III	Thyroid	8.06 (3.01-21.56)
IV	Thyroid	11.68 (4.13-33.03)
V	Thyroid	10.79 (3.66-31.83)
VI	Thyroid	18.23 (5.51-60.32)
VII	Thyroid	17.71 (5.34-58.71)
VIII	Thyroid	18.14 (5.39-61.02)
IX	Thyroid	16.78 (4.98-56.51)
X	Thyroid	17.04 (5.08-57.13)
XI	Thyroid	15.36 (4.50-52.34)
I	Uterus	0.86 (0.74-1.00)
II	Uterus	0.82 (0.70-0.96)
III	Uterus	0.83 (0.70-0.98)
IV	Uterus	0.77 (0.65-0.92)
V	Uterus	0.74 (0.61-0.90)
VI	Uterus	0.75 (0.61-0.92)
VII	Uterus	0.74 (0.60-0.91)
VIII	Uterus	0.75 (0.60-0.93)
IX	Uterus	0.73 (0.59-0.90)
X	Uterus	0.73 (0.59-0.90)
XI	Uterus	0.75 (0.60-0.92)

© 2022 Yu D et al. *JAMA Network Open*.

eTable S4. Adjusted hazard ratios for association between Pasifika (reference to NZ European) and risks of 21 cancer

Model (i) weighted for age and sex; model (ii) weighted for all adjusted variables in model (i) plus IMD group; model (iii) weighted for all adjusted variables in model (ii) plus smoking status; model (iv) weighted for all adjusted variables in model (iii) plus body measurements (body mass index, systolic and diastolic blood pressure); model (v) weighted for all adjusted variables in model (iv) plus baseline HbA1c; model (vi) weighted for all adjusted variables in model (v) plus baseline lipid profile (total cholesterol, low-density lipoprotein cholesterol, high-density lipoprotein cholesterol, and triglyceride); model (vii) weighted for all adjusted variables in model (vi) plus baseline creatinine; model (viii) weighted for all adjusted variables in model (vii) plus antidiabetes treatments; model (ix) weighted for all adjusted variables in model (viii) plus antihypertensive, lowering lipid and anticoagulant treatment; model (x) weighted for all adjusted variables in model (ix) plus entry cohorts; model (xi) weighted for all adjusted variables in model (x) plus duration of having diabetes at entry time.

Model	Cancer Type	Hazard Ratio (95% Confidence Interval)
I	Bladder	0.21 (0.07-0.60)
II	Bladder	0.19 (0.07-0.57)
III	Bladder	0.20 (0.07-0.57)
IV	Bladder	0.18 (0.06-0.55)
V	Bladder	0.19 (0.06-0.57)
VI	Bladder	0.16 (0.05-0.53)
VII	Bladder	0.18 (0.05-0.57)
VIII	Bladder	0.16 (0.05-0.55)
IX	Bladder	0.18 (0.05-0.59)
X	Bladder	0.20 (0.06-0.63)
XI	Bladder	0.21 (0.07-0.65)
I	Brain and CNS	1.48 (0.50-4.38)
II	Brain and CNS	1.55 (0.43-5.52)
III	Brain and CNS	1.65 (0.48-5.74)
IV	Brain and CNS	1.76 (0.53-5.84)
V	Brain and CNS	1.10 (0.27-4.51)
VI	Brain and CNS	1.16 (0.30-4.53)
VII	Brain and CNS	1.10 (0.29-4.23)
VIII	Brain and CNS	1.43 (0.39-5.28)
IX	Brain and CNS	1.33 (0.36-4.87)
X	Brain and CNS	1.50 (0.45-5.05)
XI	Brain and CNS	1.64 (0.49-5.46)
I	Breast	0.68 (0.49-0.93)
II	Breast	0.79 (0.54-1.15)
III	Breast	0.79 (0.54-1.14)
IV	Breast	0.79 (0.54-1.17)
V	Breast	0.79 (0.52-1.20)
VI	Breast	0.82 (0.53-1.28)
VII	Breast	0.80 (0.50-1.28)
VIII	Breast	0.87 (0.57-1.35)
IX	Breast	0.84 (0.54-1.31)
X	Breast	0.75 (0.47-1.20)

© 2022 Yu D et al. *JAMA Network Open*.

XI	Breast	0.76 (0.47-1.23)
I	Cervix	1.68 (0.53-5.36)
II	Cervix	3.10 (0.94-10.24)
III	Cervix	3.02 (0.91-9.98)
IV	Cervix	3.35 (0.93-12.03)
V	Cervix	3.00 (0.81-11.08)
VI	Cervix	3.45 (0.87-13.74)
VII	Cervix	3.75 (0.94-14.94)
VIII	Cervix	3.23 (0.78-13.34)
IX	Cervix	2.99 (0.70-12.79)
X	Cervix	3.25 (0.63-16.80)
XI	Cervix	3.38 (0.65-17.61)
I	Colon	0.40 (0.27-0.60)
II	Colon	0.37 (0.24-0.57)
III	Colon	0.37 (0.24-0.57)
IV	Colon	0.38 (0.24-0.59)
V	Colon	0.39 (0.23-0.68)
VI	Colon	0.46 (0.28-0.75)
VII	Colon	0.45 (0.28-0.73)
VIII	Colon	0.47 (0.29-0.75)
IX	Colon	0.47 (0.29-0.76)
X	Colon	0.49 (0.30-0.79)
XI	Colon	0.48 (0.30-0.78)
I	Gallbladder	12.95 (1.64-102.21)
II	Gallbladder	17.62 (2.21-140.26)
III	Gallbladder	16.92 (2.12-134.67)
IV	Gallbladder	14.99 (1.89-119.10)
V	Gallbladder	13.61 (1.71-108.44)
VI	Gallbladder	13.86 (1.75-110.00)
VII	Gallbladder	13.18 (1.66-104.61)
VIII	Gallbladder	10.21 (1.29-80.66)
IX	Gallbladder	14.53 (1.83-115.27)
X	Gallbladder	23.76 (2.97-189.83)
XI	Gallbladder	25.10 (3.14-200.63)
I	Kidney	0.40 (0.19-0.85)
II	Kidney	0.39 (0.17-0.89)
III	Kidney	0.40 (0.17-0.92)
IV	Kidney	0.35 (0.15-0.84)
V	Kidney	0.45 (0.19-1.05)
VI	Kidney	0.53 (0.23-1.22)
VII	Kidney	0.49 (0.21-1.16)
VIII	Kidney	0.53 (0.22-1.23)

© 2022 Yu D et al. *JAMA Network Open*.

IX	Kidney	0.54 (0.23-1.25)
X	Kidney	0.53 (0.22-1.29)
XI	Kidney	0.52 (0.21-1.27)
I	Leukaemia	0.60 (0.29-1.24)
II	Leukaemia	0.56 (0.25-1.28)
III	Leukaemia	0.57 (0.25-1.29)
IV	Leukaemia	0.56 (0.24-1.31)
V	Leukaemia	0.56 (0.23-1.33)
VI	Leukaemia	0.64 (0.26-1.56)
VII	Leukaemia	0.64 (0.26-1.57)
VIII	Leukaemia	0.69 (0.30-1.59)
IX	Leukaemia	0.71 (0.30-1.66)
X	Leukaemia	0.65 (0.25-1.65)
XI	Leukaemia	0.69 (0.28-1.69)
I	Liver	1.10 (0.71-1.72)
II	Liver	1.01 (0.62-1.66)
III	Liver	1.01 (0.62-1.66)
IV	Liver	1.08 (0.66-1.77)
V	Liver	1.08 (0.64-1.83)
VI	Liver	0.83 (0.46-1.50)
VII	Liver	0.85 (0.47-1.51)
VIII	Liver	0.82 (0.45-1.52)
IX	Liver	0.83 (0.44-1.57)
X	Liver	0.82 (0.41-1.63)
XI	Liver	0.84 (0.42-1.66)
I	Lung	0.96 (0.65-1.41)
II	Lung	0.77 (0.49-1.20)
III	Lung	0.78 (0.50-1.23)
IV	Lung	0.82 (0.52-1.29)
V	Lung	0.79 (0.48-1.32)
VI	Lung	0.80 (0.49-1.32)
VII	Lung	0.83 (0.50-1.38)
VIII	Lung	0.80 (0.45-1.41)
IX	Lung	0.86 (0.51-1.45)
X	Lung	0.81 (0.48-1.38)
XI	Lung	0.84 (0.50-1.42)
I	Malignant melanoma	0.01 (0.002-0.09)
II	Malignant melanoma	0.01 (0.002-0.09)
III	Malignant melanoma	0.01 (0.002-0.09)
IV	Malignant melanoma	0.01 (0.002-0.08)
V	Malignant melanoma	0.01 (0.002-0.09)
VI	Malignant melanoma	0.01 (0.002-0.10)

© 2022 Yu D et al. *JAMA Network Open*.

VII	Malignant melanoma	0.01 (0.002-0.10)
VIII	Malignant melanoma	0.01 (0.002-0.10)
IX	Malignant melanoma	0.01 (0.002-0.09)
X	Malignant melanoma	0.01 (0.002-0.10)
XI	Malignant melanoma	0.01 (0.002-0.10)
I	Multiple myeloma	0.79 (0.34-1.83)
II	Multiple myeloma	1.29 (0.53-3.12)
III	Multiple myeloma	1.31 (0.54-3.16)
IV	Multiple myeloma	1.19 (0.48-2.97)
V	Multiple myeloma	1.44 (0.59-3.51)
VI	Multiple myeloma	1.41 (0.55-3.60)
VII	Multiple myeloma	1.06 (0.39-2.86)
VIII	Multiple myeloma	1.17 (0.43-3.19)
IX	Multiple myeloma	1.19 (0.43-3.31)
X	Multiple myeloma	1.04 (0.37-2.93)
XI	Multiple myeloma	1.10 (0.39-3.07)
I	Non-Hodgkin lymphoma	0.43 (0.21-0.86)
II	Non-Hodgkin lymphoma	0.51 (0.24-1.11)
III	Non-Hodgkin lymphoma	0.51 (0.24-1.11)
IV	Non-Hodgkin lymphoma	0.49 (0.22-1.11)
V	Non-Hodgkin lymphoma	0.60 (0.29-1.25)
VI	Non-Hodgkin lymphoma	0.62 (0.29-1.31)
VII	Non-Hodgkin lymphoma	0.63 (0.30-1.33)
VIII	Non-Hodgkin lymphoma	0.57 (0.26-1.22)
IX	Non-Hodgkin lymphoma	0.60 (0.27-1.31)
X	Non-Hodgkin lymphoma	0.71 (0.33-1.55)
XI	Non-Hodgkin lymphoma	0.72 (0.33-1.55)
I	Oesophageal	0.32 (0.14-0.73)
II	Oesophageal	0.34 (0.14-0.82)
III	Oesophageal	0.34 (0.14-0.84)
IV	Oesophageal	0.21 (0.07-0.64)
V	Oesophageal	0.25 (0.08-0.79)
VI	Oesophageal	0.71 (0.39-1.29)
VII	Oesophageal	0.70 (0.33-1.51)
VIII	Oesophageal	0.70 (0.34-1.48)
IX	Oesophageal	0.70 (0.33-1.46)
X	Oesophageal	0.70 (0.34-1.48)
XI	Oesophageal	0.69 (0.33-1.45)
I	Oral cavity	0.70 (0.27-1.80)
II	Oral cavity	0.51 (0.17-1.52)
III	Oral cavity	0.52 (0.18-1.54)
IV	Oral cavity	0.51 (0.17-1.56)

© 2022 Yu D et al. *JAMA Network Open*.

V	Oral cavity	0.48 (0.13-1.73)
VI	Oral cavity	0.51 (0.13-2.01)
VII	Oral cavity	0.52 (0.13-2.16)
VIII	Oral cavity	0.45 (0.10-2.10)
IX	Oral cavity	0.58 (0.15-2.23)
X	Oral cavity	0.50 (0.12-2.01)
XI	Oral cavity	0.56 (0.14-2.21)
I	Ovaries	1.34 (0.61-2.92)
II	Ovaries	1.26 (0.48-3.30)
III	Ovaries	1.26 (0.48-3.29)
IV	Ovaries	1.19 (0.43-3.31)
V	Ovaries	1.02 (0.33-3.11)
VI	Ovaries	1.41 (0.49-4.08)
VII	Ovaries	1.45 (0.50-4.14)
VIII	Ovaries	1.66 (0.61-4.50)
IX	Ovaries	1.57 (0.57-4.35)
X	Ovaries	1.35 (0.49-3.73)
XI	Ovaries	1.37 (0.50-3.74)
I	Pancreas	0.73 (0.40-1.35)
II	Pancreas	0.57 (0.29-1.11)
III	Pancreas	0.57 (0.29-1.11)
IV	Pancreas	0.57 (0.29-1.13)
V	Pancreas	0.62 (0.30-1.28)
VI	Pancreas	0.54 (0.23-1.28)
VII	Pancreas	0.58 (0.25-1.34)
VIII	Pancreas	0.52 (0.21-1.31)
IX	Pancreas	0.51 (0.19-1.36)
X	Pancreas	0.53 (0.21-1.36)
XI	Pancreas	0.56 (0.23-1.41)
I	Prostate	0.67 (0.49-0.92)
II	Prostate	0.69 (0.48-0.99)
III	Prostate	0.68 (0.47-0.97)
IV	Prostate	0.72 (0.50-1.04)
V	Prostate	0.81 (0.56-1.19)
VI	Prostate	0.82 (0.55-1.23)
VII	Prostate	0.80 (0.53-1.20)
VIII	Prostate	0.80 (0.54-1.19)
IX	Prostate	0.79 (0.52-1.19)
X	Prostate	0.84 (0.55-1.27)
XI	Prostate	0.85 (0.56-1.28)
I	Rectum	0.35 (0.18-0.68)
II	Rectum	0.41 (0.21-0.80)

© 2022 Yu D et al. *JAMA Network Open*.

III	Rectum	0.41 (0.21-0.80)
IV	Rectum	0.40 (0.20-0.82)
V	Rectum	0.37 (0.18-0.76)
VI	Rectum	0.30 (0.14-0.64)
VII	Rectum	0.30 (0.14-0.64)
VIII	Rectum	0.27 (0.13-0.58)
IX	Rectum	0.25 (0.12-0.55)
X	Rectum	0.21 (0.09-0.49)
XI	Rectum	0.21 (0.09-0.48)
I	Stomach	1.98 (1.13-3.47)
II	Stomach	1.88 (1.01-3.48)
III	Stomach	1.89 (1.01-3.53)
IV	Stomach	1.85 (0.97-3.52)
V	Stomach	1.84 (0.92-3.66)
VI	Stomach	1.92 (0.98-3.75)
VII	Stomach	2.01 (1.03-3.92)
VIII	Stomach	1.83 (0.86-3.88)
IX	Stomach	1.95 (0.88-4.35)
X	Stomach	2.05 (0.89-4.71)
XI	Stomach	1.97 (0.84-4.63)
I	Thyroid	1.74 (0.50-6.04)
II	Thyroid	1.57 (0.40-6.07)
III	Thyroid	1.51 (0.39-5.87)
IV	Thyroid	1.90 (0.50-7.25)
V	Thyroid	2.28 (0.60-8.58)
VI	Thyroid	2.63 (0.70-9.80)
VII	Thyroid	2.73 (0.72-10.38)
VIII	Thyroid	3.69 (0.99-13.70)
IX	Thyroid	3.79 (0.99-14.47)
X	Thyroid	4.53 (1.28-16.06)
XI	Thyroid	4.47 (1.25-16.03)
I	Uterus	0.68 (0.58-0.80)
II	Uterus	0.60 (0.50-0.73)
III	Uterus	0.61 (0.50-0.73)
IV	Uterus	0.58 (0.47-0.70)
V	Uterus	0.51 (0.41-0.64)
VI	Uterus	0.48 (0.38-0.61)
VII	Uterus	0.48 (0.38-0.61)
VIII	Uterus	0.51 (0.40-0.65)
IX	Uterus	0.49 (0.39-0.63)
X	Uterus	0.54 (0.43-0.69)
XI	Uterus	0.54 (0.42-0.68)

© 2022 Yu D et al. *JAMA Network Open*.
